# Supplementary material for: The macroeconomics of abortion: A scoping review and analysis of the costs and outcomes
Source: PLoS One. 2021 May 6;16(5):e0250692. doi: 10.1371/journal.pone.0250692 (PMC8101771; doi:10.1371/journal.pone.0250692)
Supplement: S1 Appendix — (DOCX) [file pone.0250692.s002.docx]

## S1 Appendix. Summary of studies reporting macroeconomics costs (n=97)

| **Author, year [country]** | **Aim/objective(s)** | **Population** | **Study type** | **Summary of main findings** |
| --- | --- | --- | --- | --- |
| (Aantjes, Gilmoor et al. 2018) [Eastern and Southern Africa] | To identify and synthesize the literature on PAC in the ESA region with the aim of reporting on the reach, quality and costs of these services | Varied by studies on PAC, health workers in PAC, incidence and abortion-related service reviews | Systematic review of studies on PAC | Treatment costs for abortion morbidity represented three times the annual per capita health expenditure in Uganda and five times that in Ethiopia. |
| (Adamczyk and Valdimarsdóttir 2018) [United States] | Examines the role of the local religious context for shaping attitudes and the presence of a county abortion clinic | U.S. residents 18 years of age or over | Regression analysis | Since 2011 the number of abortion clinics in the US has declined, which some have attributed to TRAP's effectiveness, as well as other factors like fewer unintended pregnancies. As the level of county religious engagement rises, religious and secular residents alike develop more conservative attitudes. Conversely, as the county Catholic rate increases, moderate and liberal Protestants become more prochoice. While the county conservative Protestant rate has no influence on residents' attitudes, it is the only religious contextual measure that shapes the likelihood that a county has an abortion clinic. |
| (Agadjanian 2002) [Kazakhstan] | This study shows how abortion-related views reflect the long-standing ethnocultural differences between the indigenous Kazakhs and Kazakhstan’s residents of European roots | Women in Kazakhstan | Regression analysis | The bivariate comparisons suggest that for all three groups, abortion is associated with greater financial problems and especially with greater health problems than either the IUD or the pill. At the same time, non-Russified Kazakhs appear more concerned about financial costs of any of the three choices, whereas Europeans seem particularly preoccupied with potential negative side effects to health of abortion and, to a much lesser degree, of contraception. Indicators of greater modernization and affluence are inversely associated with the perception of monetary costs as a problem. |
| (Almond, Edlund et al. 2013) [Canada] | The study of South and East Asian immigrants in Canada, a rich OECD country, can potentially cast light on the role of culture in sex selection | Census data from Canada | Descriptive statistics | Since the overwhelming majority of Asian immigrants live in large cities, however, access to facilities can be assumed for the population under study. Although cost is another consideration, abortions in Canadian hospitals are covered by the universal public health plan in every province and territory, as are abortions in private clinics almost everywhere. |
| (Ananat, Gruber et al. 2009) [United States] | Provide a framework for understanding selection mechanisms and use that framework to address inconsistent past methodological approaches and provide evidence on the long-run impact on cohort characteristics | Individuals born in the United States and observed in the 2000 Census at ages 21 to 35 in that year (born between 1965 and 1979) | Regression analysis | Results indicate that lower-cost abortion brought about by legalization altered young adult outcomes through selection. In particular, it increased likelihood of college graduation, lower rates of welfare use, and lower odds of being a single parent. Higher abortion costs reduce the pregnancy rate: non-repeal states had lower pregnancy rates during the 1971–1973 period; among non-repeal states, those with higher latent social costs of abortion experienced lower pregnancy rates. The same pattern continues in 1974–1975 and 1976–1979. As expected, the negative relationship between latent costs and the pregnancy rate becomes more pronounced after legalization: once the legal constraints are removed, the underlying latent costs of abortion become the primary determinant. Higher abortion cost predicts higher birth ratios (or equivalently, fewer abortions) and higher birth rates. |
| (Ayanore, Pavlova et al. 2017)[Ghana] | To explore what direction do women perceive gender roles to influence their reproductive choice and care | Data from women with records of recent birth (max 2 years prior to study) (n=90), health staff (n=16), policymakers (n=6) | Qualitative ethnographic study using focus groups and in-depth interviews | Policymakers responded that post-abortion fee costs and clinical treatments for post-abortion care were minimally covered under fee exemptions. |
| (Azize-Vargas and Avilés 1997) [Puerto Rico] | This paper examines the current practice of abortion and the hurdles women face to obtain this service, taking into consideration the impact of colonial subordination | Women attending 10 of the 13 private abortion clinics in Colombia 1991-2 | Mixed methods | Lack of government and health professional support has meant that about 93 per cent of all abortions in Puerto Rico are carried out by individual doctors in private, profit-making providers. The absence of any public health or other non-profit abortion services means that there is little motivation to keep prices down. The US funded program Medicaid is supposed to cover the costs of abortion in cases of rape, incest and when a woman’s life is in danger. Although the Puerto Rican government receives Medicaid funding, abortion is not covered, even in these extreme cases. Since private health insurance does not include abortion among its benefits either, a woman must attend a clinic with cash in hand when she needs an abortion. Hence cost is a barrier for obtaining abortions in Puerto Rico for poor women. |
| (Babigumira, Stergachis et al. 2011) [Uganda] | Perform a comprehensive assessment of the economic burden of induced abortion in Uganda in terms of its costs | Providers, untrained providers, and abortion-seekers | Cost analysis | The average societal cost per induced abortion (95% credibility range) was $177 ($140–$223). The average direct medical cost was $65 ($49–$86) and the average direct non-medical cost was $19 ($16–$23). The average indirect (productivity) cost was $92 ($57–$139). Patients incurred an average of $62 ($46–$183), 73% of the healthcare costs of induced abortion while government incurred an average of $14 ($10–$20), 17% of the healthcare costs of induced abortion. The annual incidence of induced abortion in Uganda is 362,000 cases. Therefore the national annual expenditure on induced abortion is projected to be $23.6 million in direct medical costs, $7.0 million in direct non-medical costs, $33.5 million in indirect/productivity costs, $5.1 million in costs to the government, $22.3 million in costs to patients, and $64.2 million in societal costs. |
| (Baird 2015) [Australia] | This paper considers the context through which it (medical abortion) has become available (in Australia) since 2013 | Documentary sources, secondary literature, and expert opinions from health professionals and managers who have worked in abortion services, researchers, educators, public servants, advocates and activists | The paper draws on documentary sources and oral history interviews conducted in 2013 and 2015. | In the context of the ongoing absence of any central public health coordination of abortion services in all jurisdictions, and in the face of the slow uptake of medical abortion by general practitioners, telemedicine stands out as a method of materializing access, particularly for rural women. Marie Stopes have started a modest trial to offer medical abortion via telemedicine but the most significant development in this respect has been the launch of the Tabbott Foundation in September 2015. This new body pioneers an innovative business model for abortion provision. It has been established by an existing private provider to offer “an Australia-wide telephone consultation home medical termination of pregnancy service”, although women in the jurisdictions which require abortion to be performed in a hospital will not be served.38 Women in rural and regional locations are the foundation’s main focus. |
| (Baird 2017) [Australia] | Argues that while decriminalization is consistent with feminist goals, it will make little or no challenge to the sources of inadequate access to abortion in Australia | Documentary evidence and an oral history project in 4 Australian jurisdictions | Review of documents and oral history interviews conducted with key insiders | The nation has a comprehensive public health system, albeit one that struggles under the trends and pressures characteristic of neoliberal policy approaches. Australia has a strong tradition of social democracy but at the level of everyday cultural practice and of government social and economic policy, individualism, choice, and market-based solutions in all areas of life have become common sense. The role of the state is to facilitate markets.4 Health is understood largely as an individual responsibility, and the public health system (including public hospitals) negotiates competing principles in the context of ongoing pressure to privatize, limited resources, and constant restructuring. |
| (Battistelli, Magnusson et al. 2018) [United States] | Qualitative analysis examines how organizations participating in HWPP implemented changes to their provision of abortion care following the law’s implementation in California | Health providers employed by six organizations located in California | Interviews | Several respondents cited the state’s low Medicaid reimbursement rate for abortion services as a key driver of the maldistribution of care. Instead of trying to spread abortion services throughout the regions they serve to make care more geographically accessible, some organizations had consolidated such services into just a few high-volume clinics to be economically sustainable. |
| (Benson, Gebreselassie et al. 2015) [Malawi] | This study estimates current health system costs of treating unsafe abortion complications and compares these findings with newly-projected costs for providing safe abortion in Malawi | Malawi health system costs | Estimation study based on survey and costing data | Within the study facilities, the estimated median per-case cost of treatment for all PAC cases was $40 The median cost per D&C case ($63) was 29 % higher than an MVA case ($49) for every PAC treatment category. This difference was especially marked for simple cases as the median cost of care for a simple PAC case with D&C, $19, was 46 % higher than with MVA ($13) and 58 % higher than misoprostol ($12). Although few in number, cases with severe surgical complications were especially costly to treat ($128), almost 10 times higher than a simple case. |
| (Berer 2000) [Global] | This article examines the changes in policy and health service provision required to make abortions safe | It is based on a wide-ranging review of published and unpublished sources. | Review | Where abortion is clandestine and unsafe, women (or their partners and families) are buying drugs and other means of self-induced abortion and/or paying clandestine providers, while public health services and women are paying for the treatment of abortion complications, often in tertiary level hospitals, where costs are highest. Unsafe abortion situations are characterized by a lack of equity in cost, safety and quality of care. Covering the cost of safe abortions in public health services is therefore not about incurring entirely new costs, but about shifting expenditure away from complicated cases in tertiary level hospitals to safe, simple procedures which can be provided in primary clinics. Women may or may not be charged a fee at the point of service, but safety means affordability for the poorest of women as well as for those who can pay, with one high standard of care for all. |
| (Berer 2005) [Global] | This paper discusses choice and acceptability of medical abortion from the perspective of both women and abortion providers and argues that choice of method is important for both | Recent papers found on Medline on these issues from a range of countries, and information presented at a 2004 conference on medical abortion | Review | Misoprostol alone is very low cost and has ease-of-access advantages for developing country settings. According to most of the developing country participants at the ICMA conference in 2004, misoprostol is likely to be used on its own for early medical abortion in settings where registration of mifepristone in the foreseeable future is unlikely. On the other hand, as one clinician from Latin America noted, if women take repeat doses of misoprostol, it may end up being more expensive than one 200mg pill of mifepristone and a smaller dose of misoprostol. Similar cost reduction studies for surgical methods are needed. Unless the costs of surgical and medical abortions are broadly similar, many women will feel they need to choose the cheaper option, which limits choice. Where the health service is covering the cost, or health insurance companies control treatment options, similarly, clinicians and managers will also tend towards the cheaper option. However, neither method should be forced on women for this reason. At the same time, because medical abortion does not require the use of an operating theatre or surgical skills, except in a small minority of cases, thereby drastically reducing the number of clinicians with these skills involved, all health systems may tend towards medical abortion in future. |
| (Bessett, Gorski et al. 2011) [United States] | The aim is to learn about women’s experiences applying for subsidized insurance and to identify barriers to obtaining insurance or its use for abortion services | Women of childbearing age in Massachusetts. | Interviews | Applying for subsidized insurance was also associated with long delays. Although pregnant women’s applications are supposed to be expedited, it often took several weeks to become enrolled even if everything went smoothly. Unnecessary delays should also be a matter of concern to policy makers in Medicaid states, because procedures at later gestational ages are more expensive and delays may increase costs to the state. |
| (Blanchard, Meadows et al. 2017) [United States] | Studied women's experiences seeking and receiving second-trimester abortion care in two geographically and legislatively different settings to inform ways to improve abortion care access and services | Women with estimated gestational age of ≥14.0 weeks LMP; age 18 or older; English fluency; ability to give informed consent | Mixed methods: descriptive statistics and qualitative | Interviewees generally opposed all restrictions, especially Medicaid restrictions, saying they failed to consider individual circumstances and infringed on the ability to make one's own decisions. Opinions about bans on abortions 22 weeks LMP, restrictions on Medicaid coverage, and mandatory waiting periods did not vary significantly between regions. There were differences in opinions about mandatory counseling: almost half of the women in the Midwest versus less than a third of the women in the Northeast disagreed with the law. Even if the information was inaccurate, more women in the Northeast than the Midwest still agreed with the law. More participants reported being negatively impacted by abortion restrictions than being positively impacted. Commonly cited negative impacts included delayed care, significant travel, and having to take work leave. The most commonly cited positive outcome was feeling more informed. |
| (Blank, George et al. 1996) [United States] | This paper investigates the impact of public policy, provider availability, the political environment, and demographic and economic factors on the determinants of state abortion rates between 1974 and 1988 | Number of abortions, abortion-related legislations | Regression analysis | Restrictions on Medicaid funding for abortion are strongly correlated with decreases in the abortion rate within states and with increased travel of women across states for abortions, while restrictions on teenagers have little effect on aggregate rates. |
| (Brown and Jewell 1996) [United States] | To estimate directly the responsiveness of abortion demand to county-level variations in travel-cost component of the full cost of abortion services | Abortion providers in Texas | Log-linear regressions using data that were obtained from health facilities on each abortion performed and data on the localities of these facilities | Counties with providers have higher time costs, due to higher median household incomes. |
| (Bullard, Shaffer et al. 2018) [United States] | To estimate the effect of 20-week abortion bans on maternal and consequent neonatal health outcomes and costs in the setting of fetal congenital diaphragmatic hernia | Women in their mid-second trimester of pregnancy with a prenatal diagnosis of congenital diaphragmatic hernia | Cohort analytic | With a 20-week abortion ban in place, 60 women traveled out of state to obtain an abortion. In terms of cost, having a 20-week abortion ban in place cost an additional $159,419,623 for this cohort of 921 women. This additional cost came with a reduction of 674 QALYs. Overall, 20-week bans were a dominated strategy; the policies resulted in higher costs and decreased effectiveness, thereby deeming the absence of a 20-week ban to be the optimal strategy. |
| (Calkin 2019) [Global] | This article makes the case for a political geography of abortion that moves beyond a state-based framework to account for changing patterns of resistance and restriction on abortion | Two case studies: mobile abortion clinics at sea and telemedicine abortion technology | Case study, literature review | Abortion restrictions continue to close clinics and restrict their operation: for example, there are twenty-seven cities in the US that qualify as “abortion deserts” because their nearest clinic is over 100 miles away. In practice, these geographical barriers become class-based obstacles: the absence of public transport in rural areas, for instance, means that travel to a far-away clinic requires an abundance of money and time that poor and rural women disproportionately lack. |
| (Chevrette and Abenhaim 2015) [United States] | Assess whether US state-level policies regarding  abortion and sexual education are associated with different teen birth and teen abortion rates | National teen birth and teen abortion rates | Regression analysis | In the states where deterrents to abortion were present, the teen abortion rate was 11.7 of 1000, compared with 19.1 of 1000 in the other states. When access to public or private insurance was prohibited for abortions (or limited to cases of rape or a woman's life being endangered), teen abortion rate was 13.8 of 1000 compared with 18.8 of 1000 in the other states. |
| (Coast, Norris et al. 2018) [Global] | Present a new conceptual framework for studying trajectories to obtaining abortion-related care | Global review paper | Review | A pregnancy has short- and long-term economic and opportunity costs for women; these may be exacerbated when the pregnancy is unintended. In Latin American countries where abortion is illegal, access to economic resources and emotional support were critical for accessing a medically supervised medical abortion in a clandestine clinic. |
| (Coles, Makino et al. 2010) [United States] | Examine the relationship between adolescent pregnancy intention and policies affecting abortion access: mandatory waiting periods, parental involvement laws, and Medicaid funding restrictions | Women under 18 years of age at 3 months gestation | Bivariate analyses | Those living in states with either Medicaid funding restrictions or mandatory waiting periods reported higher percentages of both unwanted and mistimed birth compared to minors living in states without these statutes. Black and Hispanic teens were most strongly affected by mandatory waiting periods. These findings likely reflect resources availability more than cultural differences, as minors with Medicaid were also more likely to report an unintended birth when exposed to mandatory waiting periods. |
| (Cook 1999) [United States] | To estimate the funding effects on abortion rates and birth rates | Women who received abortions in North Carolina and data on the number of pregnancies | State level data analysis | In financial year 1989, 60% of people receiving a state-funded abortion were aged between 18 and 25. 75% were Black and 80% were unmarried. An additional 16% were separated or divorced. Overall, 7.1% of pregnant Black people received state funds for their abortion, whereas 1.1% of pregnant White people received state funds for their abortions. |
| (Creinin 2000) [United States] | To examine in a randomized trial the clinical efficacy and patient acceptance of medical abortion using oral methotrexate and vaginal misoprostol compared to surgical manual vacuum aspiration in women with pregnancies up to 49 days’ gestation. | People aged 18 years and older requesting to have an abortion (n=50) | Randomized trial [no control] | The estimated staff costs in different provider scenarios. Based on a physician’s hourly wage being 3 times that of a physician assistant and 8 times that of a research assistant / counselor, the estimated that surgical abortion would be 10% more expensive than medical abortion.  However, where the physician’s hourly wage is 2 times that of a physician assistant, surgical and medical abortion costs are identical. |
| (Crighton and Ebert 2002) [Europe] | Explore the impact of RU486 on abortion rates and practices in Europe | Abortion rates, policies, and practices for each nation state | Policy analysis from published literature | Finds no evidence that access to medical abortion with RU 486 has increased abortion rates in Europe. Study does find large national differences in the use of medical versus surgical abortions. These abortion practices are shaped by economic incentives, the organization of medical services, bureaucratic accounting rules, and physicians' choices. Effective implementation, with governmental support, has a critical impact on access to medical abortion services. |
| (Davey 2005) [United Kingdom] | To present results of a review of the sexual health situation in the UK and to outline the challenges remaining in order for the UK to meet ICPD goals by 2015 | UK residents | Situational review | Access to abortion services has improved in recent years. In 2003, 80% of abortions in England and Wales were funded by the NHS (National Health Service) compared with 67% in 1994. The vast majority (87%) were carried out under 13 weeks of pregnancy. In Scotland, in 2003, 99% of abortions were NHS-funded, and 92% were carried out within 13 weeks. However, within England, Wales and Scotland, access to an NHS abortion varies considerably according to where an individual lives. 2002 figures show that while in some Primary Care Trust areas almost all abortions were NHS-funded, in others less than two-thirds were. |
| (Dawson, Bateson et al. 2016) [United Kingdom, United States, Russia, Australia, New Zealand, Canada] | To identify quality studies of abortion services to provide insight into how access to services can be improved in Australia | Systematic review with included studies looking at multiple populations | Systematic review | Some of the studies included in this review examined costs from the perspective of the health system, health professionals and the individual women. For example, recent research in the UK examining a local anesthetic outpatient STOP service found that a cost savings was made of approximately £60,000 per year and that the operating theatre use was reduced by one termination list per week. |
| (de Bruyn 2003) [Global] | Conduct literature review on unwanted pregnancy and induced abortion among women with HIV | women living with HIV | Literature review | This paper describes the difficulties faced by HIV-positive women in obtaining safe legal, affordable abortion services. It shows that voluntary HIV counselling and testing for women seeking induced abortions and post-abortion care may not be provided. HIV-positive women may have a greater risk of morbidity following unsafe abortions than HIV-negative women. Studies in Zimbabwe and Thailand show that when information and access to legal pregnancy termination are lacking, HIV-positive women may be prevented from terminating a pregnancy. |
| (Dennis, Manski et al. 2015) [United States] | Explore low-income women’s experiences accessing abortion in Massachusetts | Low-income women age 18 or older, have had an abortion after January 2009, and, at the time of the abortion, resided in Massachusetts | Interviews | Most women described having access to timely, conveniently located, affordable, and highly acceptable abortion care. However, a sizable minority of women had difficulty enrolling in or staying on insurance, making abortion expensive. Some limited data also suggest that young women and immigrant women face specific barriers to care. There is a need for state-level policies that support access to health insurance and comprehensive abortion coverage. Such policies, along with a well-functioning health care environment, help to ensure that low-income women have access to abortion. |
| (Dennis, Manski et al. 2014) [United States] | Address 1) What do women know about the cost of abortion and the availability of Medicaid coverage for abortion? 2) Where do women obtain this information? and 3) What are women’s experiences paying for care? | Low-income women age 18 or older, have had an abortion within the past two years, and resided in one of the four study states at the time of the abortion | Interviews | Women's impressions about abortion costs and the availability of Medicaid coverage are generally accurate and that women rely predominantly on abortion facilities for confirmatory cost and coverage information. Additionally, when abortion is out of financial reach, women and the people in their lives experience numerous emotional and financial harms. Policies that aim to ensure abortion is affordable largely prevent these harms, though the availability of Medicaid coverage does not always guarantee access to affordable care. |
| (Díaz-Olavarrieta, Cravioto et al. 2012) [Mexico] | Identify the perceptions and opinions of people who provide abortion services in Mexico City, three years after implementation of elective abortion legal reforms | Health workers assigned to the legal abortion programs at a clinic and a hospital in Mexico's Federal District | Interviews | Participants favored the abortion law for the rest of the country to replicate the reforms made in the Federal District as this would prevent mobilization of women from other states, which is very expensive for them and increases the workload in the services. To this respect, from all abortion procedures up to March 2010, 25% corresponded to women who reside in other states. |
| (Donohoe 2005) [United States] | To present barriers to abortion in the US policy context, and the impact of US policies on abortion access elsewhere | US abortion-related policies | Descriptive study of barriers to abortion | Title X Family Planning Clinics cover women in low-income households, and predominantly serve BIPOC, but are prohibited from using federal and non-federal funds for any abortion. |
| (Drovetta 2015) [Latin America & Caribbean] | Describes the implementation of five Safe Abortion Information Hotlines (SAIH), a strategy developed by feminist collectives in a growing number of countries where abortion is legally restricted and unsafe | Participatory observation of activities of the SAIH, and in-depth interviews with feminist activists who offer these services, and with 14 women who used information provided by these hotlines to induce their own abortions | Qualitative study: participant observation, interviews, and review of documents | In general, the hotline constitutes an easily accessible tool, especially in urban contexts, for anyone with a landline or cellphone. If a woman cannot cover the cost of the call, she can ask to be called back, leaving her number. With a cellphone, the first contact can be made via text message. |
| (Duggal 2004) [India] | Examination of the political economy of abortion care in India by reviewing cost and expenditure patterns for abortion care in India | Population of India | Descriptive analysis of the political economy | In the public sector, abortion services are usually free, but in recent years some states have introduced user fees or have allowed private practice by public providers. Hence, such charges were being reported in the six-state study. Further, in some states, even if abortion services per se are free, there is a policy of charging for the abortion if a family planning method is not also accepted by the woman. Overall in the six states, the average charges for an induced abortion were Rs.615. This is equivalent to more than three weeks of average per capita income for all- India. The overall charges in the public sector averaged Rs.115 (or four days of per capita income) and in the private sector Rs.801 (or 30 days of per capita income). Public providers were the least expensive, and among the private providers, the certified ones were charging substantially higher fees than the uncertified ones. |
| (Duggal and Ramachandran 2004) [India] | To synthesize findings and reports from the Abortion Assessment Project – India | 380 facilities in six states in India | Review of studies: policy reviews, multicentred facility surveys, 8 qualitative studies, community-based studies | The abortion economy was estimated as worth Rs. 6,950 million (154 million USD) in 2001, which is 0.58 of the total out-of-pocket expenditure for health care |
| (Dzuba, Winikoff et al. 2013) [Latin America & Caribbean] | Present evidence of medical abortion (MA)’s contributions to reduced complications, describe strategies to enhance safe MA, and highlight existing barriers to access in LAC | Women seeking abortion in Latin American and the Caribbean | Review of existing evidence | Medical abortion conserves the resources of public healthcare systems in treating serious and largely preventable complications. An estimated US$94.00 per-patient cost to treat post-abortion complications in LAC implies an annual cost of US$108,000,000 to healthcare systems throughout the region. The cost of treating abortion complications could exceed 50% of a healthcare system’s expenditures on all obstetric emergencies. In 2005, in Mexico City, before first trimester abortion became legal, this cost to the system was estimated to be US$2.6 million. Ensuring access to safe medical abortion reduces abortion complications and allows scarce resources to be directed to other urgent health needs. |
| (Ely, Hales et al. 2017a) [United States] | Use a trauma-informed lens to explore abortion-related hardships in a previously understudied group | Status as abortion seeker and inability to pay for abortion care procedure | Descriptive, exploratory, cross- sectional analysis of administrative health care data | Patients from states that do not use state Medicaid funds to exceed coverage of abortions (outside of instances of rape or incest) had a higher average reporting of hardships related to abortion and abortion care. This ﬁnding was also true for patients from states that restrict the private insurance coverage of abortion, with patients from these restrictive states experiencing a higher average number of hardship experiences. This is also not surprising, given that states must elect to use their own Medicaid funds to cover the procedure, and the states not covering it are most often located in the South or Midwest, where the greatest numbers of hardships were found. Even when these states should be covering abortion in cases of rape or incest, problems getting the procedure paid for by Medicaid are common. |
| (Ely, Hales et al. 2017b) [United States] | Discuss the results of a secondary data analysis of NNAF’s Tiller Memorial Fund cases that represent patients who received funding pledges to assist them with paying for an abortion | Status as abortion seeker and inability to pay for abortion care procedure | Descriptive, exploratory, cross- sectional analysis of administrative health care data | Patients in the current study on average received almost $250 per case in NNAF funding assistance pledges and almost $800 in additional funding assistance from other sources. This extensive funding assistance from various sources demonstrates the significant impact of NNAF and the other funding organizations, and highlights the tremendous financial need that these women were facing when trying to access an abortion. This finding also highlights the effort these patients had to put into piece together the funding for an abortion procedure from more than one source, and the findings demonstrate the public health costs that are being picked up by these advocacy-oriented organizations. Perhaps most importantly, results from the current study are the first to demonstrate that funding pledges for second trimester abortions increased over time. This demonstrates that more women are seeking to fund procedures in the second trimester, which are more expensive and involve greater risk. This increased need for funding for second trimester procedures suggests that it may be becoming increasingly more difficult for women to gather the funding needed early enough to obtain the procedure in the first trimester. |
| (Ely, Hales et al. 2017c) [United States] | Examine Location and Travel Distance in U.S. Abortion Fund Patients | Status as abortion seeker and inability to pay for abortion care procedure | Descriptive, exploratory, cross-sectional analysis of administrative health case data | These findings suggest that patients with pledges from states restricting private insurance coverage expected to travel greater distances than patients from non- restrictive states. Restricting insurance coverage forces individuals to procure abortion costs privately. For low-income persons, obtaining costs for a procedure may take weeks or months, forcing patients to travel greater distances to providers who offer services for late stage procedures. However, contrary to expectations, patients in Medicaid expansion states expected to travel further distances than those in non- expansion states. The reason for this finding needs to be explored in future research, but a possible explanation may be that states in this region are larger (i.e. California) which results in the need to travel farther to access the procedure. Another possibility is that the expansion states have higher numbers of rural patients, who must travel greater distances to access providers, since 89% of U.S. counties do not have an abortion provider. |
| (Ely, Hales et al. 2018) [United States, Republic of Ireland, Northern Ireland, Isle of Man] | Compare abortion fund patient experiences across these developed nations for the first time | Selected abortion funds in each country | Cross-section descriptive analysis | The current study suggests that abortion fund use is increasing yearly across these nations and that abortions are costly and difficult to pay for. The assistance given by these abortion funds is an essential piece in bridging the gap to abortion access across these nations. While existing policy continues to ban or restrict abortion in these nations, steps should be taken to promote the resources offered by abortion funds and to increase financial support for abortion funds. |
| (Erim, Resch et al. 2012) [Nigeria] | Synthesize the best available data, adapt a model of pregnancy and pregnancy-related morbidity and mortality to the Nigerian context, and conduct national and regional analyses that quantify the payoffs from investing in safe pregnancy and childbirth | National level estimates | Mixed methods | Estimates of costs under current standard of care ( 2008 US$ ): post-abortion complications (US$50.73); elective abortion (US$21.87) |
| (Felkey and Lybecker 2014) [United States] | Measure whether young women are less careful in using contraception if abortions are less costly, both in the context of ﬁnancial and opportunity costs. | Women under the age of 25 | Regression analysis | The effects of abortion restrictions for minors are largest and the most significant for women aged 18 and younger, and the effect of these restrictions decrease in magnitude and significance gradually as women age. As the percent of the state's women without a provider increases, abortions are more difficult to obtain, and women are more likely to use the pill. When a larger percentage of women have a provider, abortions are more easily obtained, and there is a negative effect on pill usage. These results indicate that young women are forward thinking when making their contraceptive decisions, relative to the direct and indirect restrictions on abortion access. |
| (Felkey and Lybecker 2018) [United States] | Analysis of state abortion legislation and proxying how the cost of obtaining an abortion varies across states, then assessing the implications of legislative changes on women’s contraceptive choices | US women making observable contraceptive choices | Regression analysis | Examining women by race/ethnicity, income, age, and religious affiliation, the results show that women respond to increased restrictions on abortion availability and cost but that the effects are very small. This study demonstrates that legislation restricting women’s access to abortions fails to promote greater use of more effective contraceptive methods, increasing the likelihood of unwanted births and illegal abortion procedures. |
| (Fischer, Royer et al. 2018) [United States] | Seek to understand the impact of family planning and abortion clinic access on abortions, births, and contraceptive purchases | Women in designated Texas counties | Regression analysis | Findings suggest that restrictions in abortion access have economically-significant effects on fertility-related outcomes. Having no abortion provider within 50 mi reduces the observed number of abortions by 16.7%. Although the estimate may not capture the effect on the total number of abortions, the estimates suggest that these policies increase the cost of seeking an abortion. It is possible some women may travel to another state or country for an abortion or self-administer one (i.e., they receive the abortion they intended to obtain). These actions can be costly and thus, should be considered part of the burden of these policies.9 For this reason, the impact of the reduction in abortion access on births, a 1.3% increase, is more informative of the total effect on fertility-related behaviors. The effect of reduced family planning access on births, as measured by whether or not there is a funded clinic within 25 mi, is similar. Overall, not having a funded clinic within 25 mi increases births by 1.2%. Back-of-the-envelope calculations imply that by the end of 2015, the abortion clinic restrictions led to 1570 additional births and the changes in funding to family planning clinics increased births by 929. These calculations, however, miss other important costs of reduced access — most importantly, the increased travel cost for women seeking abortion or family planning services. |
| (Forrest and Samara 1996) [United States] | To estimate the annual numbers of unplanned pregnancies, births and abortions averted by use of publicly funded family planning services in the United States | Women using contraception who had recently visited a publicly funded family planning service provider | Analysis of 4 hypothetical scenarios of contraceptive behavior using survey data | The public sector spent an estimated $412 million on publicly funded contraceptive services for FY1987. In contract, according to the average estimates from Scenarios I-III, if publicly funded services were not available, the federal and state governments would spend an additional $1.2 billion annually in their Medicaid pro- grams to cover costs associated with un- planned births ($1.2 billion) and abortions ($22 million). Thus, for every dollar spent to provide publicly funded contraceptive services, the public saved an average of $3.00 on Medicaid costs for pregnancy-related and newborn medical care. Using the gross numbers of pregnancies, births and abortions averted from Scenario IV, the paper arrives at savings of $7.80 per dollar spent. |
| (Foster, Jackson et al. 2008) [United States] | Examine and report on factors associated with abortion delay | Female patients at the San Francisco General Hospital Women's Options Center | Secondary data analysis of a cross-sectional study | Difficulty with getting MediCal to pay for the abortion was significantly associated with delay during the second step of the abortion process. Several factors may contribute to difficulty with getting MediCal to pay for the abortion including women's lack of knowledge about available coverage, difficulty negotiating the MediCal application process or difficulty locating an abortion provider that accepts the MediCal payment. Although the state Medicaid program covers abortion care for poor women, not all providers accept that coverage and even those who accept MediCal do not accept it for abortions at all gestational durations. California is unusual in its public funding of abortion. Thirty-seven states ban Medicaid funding for abortion unless the pregnancy is a result of rape or incest or poses a risk to the woman's life. Medicaid- eligible women in other states could face longer delays as they seek to finance their medical care with their own funds. In the step between calling a provider and obtaining the abortion, many women report being delayed by financial factors. Reducing the delay related to an inability to pay could be carried out through interventions aimed at helping women finance their abortions. These interventions may include grassroots efforts like expanding the network of abortion funds which currently provide support for low- income women or policy efforts like working to increase private and public insurance coverage of abortion. A final intervention may be encouraging abortion clinics located in states where Medicaid covers abortion to accept Medicaid for abortions at all gestational durations. |
| (Foster and Kimport 2013) [United States] | To analyze data on women who sought and received an abortion at or after 20 weeks’ gestation for reasons other than fetal anomaly or life endangerment | People who were seeking abortions after 20 weeks gestation across 16 sites (n=272) and people who presented for first trimester abortions (n=169) | Mixed methods – qualitative data from interviews and quantitative data for logistic regression | Medi-Cal, the California Medicaid program, covers abortion costs for women with low-incomes.  Delays in coverage leads to later abortion and increased costs. |
| (Franzini, Marks et al. 2004) [United States] | To assess the potential economic costs that result when adolescents do not seek reproductive health care services because their conﬁdentiality is compromised | Adolescents in Texas | Regression analysis | Reporting and consent requirements were estimated to result in an additional 11.45 pregnancies, 7.44 births, and 2.29 abortions per 100 teenagers currently receiving reproductive health care services. The cost of the additional births and abortions was estimated at $60 952 per 100 teenagers. Among Texas girls younger than 18 years currently receiving publicly funded reproductive health services, an estimated 5372 additional births and 1654 additional abortions cost $44 007. |
| (French, Anthony et al. 2016) [United States] | To assess the association of clinician referral with decision-to-abortion time. | English-speaking women aged 19 years and older presenting for an abortion for all indications at the three abortion clinics in Nebraska [n=263] | Cross-sectional survey | Most abortions in the United States are paid for out of pocket (57%) or Medicaid coverage (20%). In Nebraska and 31 other states, Medicaid funds may be used to pay for abortion only in cases of life endangerment, rape or incest. Consequently, even more women pay for their abortions out of pocket, creating hardships for themselves and their families |
| (Furedi 1999) [United Kingdom] | Estimate the effect of increased unintended pregnancies, births and abortions after the 1995 birth control "pill scare" | UK population | Review study | The unanticipated increase in births and abortions led to increased expenditure on maternity and abortion services. Answers to Parliamentary Questions have recently confirmed the cost to the National Health Service (NHS) of a first-trimester abortion to be between £289 and £443 and the average cost of childbirth to be £1698. If, as suggested in para. 3.4 of the Hansard document, there were about 12 400 additional births and 13 600 additional abortions (70% paid for by the NHS) in 1996, the cost to the NHS would have been about £21 million for maternity care and from £46 million for abortion provision. In the long term there will be continuing costs to the Government from social security payments and from the provision of extra school places. |
| (Gerber Fried 1997) [United States] | This paper gives a picture of the status of legal abortion from the vantage point of those women who bear the brunt of restricted access | Low-income women, women of color (who comprise a disproportionate number of the poor), and young women | Review | Within the system of privatized health care in the US, a large majority of abortions are paid for by the patients themselves. About one-third of women do not have employment-linked health insurance. One-third of private plans do not cover abortion services, or only cover these for certain medical indications. At least 37 million Americans have no health care coverage at all, including nine million women of childbearing age. Abortion is the only reproductive health care service for which Medicaid does not pay. Medicaid is a publicly funded program that covers ‘necessary medical services’ for people whose combined income and resources are considered insufficient to meet the costs of medical care. However, because the eligibility ceilings are set so low, Medicaid covers fewer than half of those who live in poverty. Federal Medicaid coverage was available for abortion from the time that state-level abortion laws began to be liberalized in the late 1960s until 1977, four years after Roe v. Wade made abortion legal nationwide. Since then, each year the US Congress has passed different versions of the Hyde Amendment, which prohibits federal funding of abortion. |
| (Gerdts, DeZordo et al. 2016) [United Kingdom] | Better understand the experiences of non-resident women who travel to the UK seeking abortion services | 58 non-UK residents seeking abortions at three British abortion clinics | Small-scale survey | Abortion travel represents a financial cost that is difficult to cover for many women. It is well known that travel in pursuit of health care services creates problematic cost, access and resource distribution in health care systems, and stratifying consequences for populations and individuals. The issue of cross-country abortion care is especially significant considering the 2013 EU Cross-Border Health Directive which would allow women to receive abortions abroad and be reimbursed by their home nations. |
| (Gober 1997) [United States] | Investigate the role of access in explaining the variation in state abortion rates | U.S. state policies and abortion rates | Regression analysis | Greater accessibility leads to higher abortion rates. Public demand variables affect abortion rates both directly and indirectly through access conditions. The number of women at risk of unintended pregnancies leads to higher abortion rates directly and indirectly through its effects on medical access. Per capita income, percent Catholic, and percent of the population born outside the state affect abortion rates indirectly through the access variables. High per capita income leads directly to greater availability of hospital abortions, higher levels of state funding of abortions for poor women, less restrictive state abortion laws, and indirectly to higher abortion rates. States with large non-native populations have less restrictive abortion laws and higher abortion rates. The presence of a large Catholic population reduces the number of hospitals offering abortion services and leads indirectly to lower abortion rates. The interaction of public demand and access at the state level creates geographically varying environments in which abortion decisions are made. |
| (Goldie, Sweet et al. 2010) [India] | Estimate the clinical and population-level benefits associated with strategies to improve the safety of pregnancy and childbirth in India | Country- and region-specific data were synthesized | The best available data were synthesized using a computer-based simulation model | An integrated and stepwise approach (increased family planning and safe abortion combined with consecutively increased skilled birth attendants, improved care before and after birth, reduced home births, and improved emergency obstetric care) could eventually prevent nearly 80% of maternal deaths. All the steps in this strategy either saved money or involved an additional cost per year of life saved of less than US$500; given one suggested threshold for cost-effectiveness in India of the per capita GDP (US$1,068) per year of life saved, these strategies would be considered very cost-effective. While the initial strategy was cost saving in both urban and rural India, incremental cost-effectiveness ratios ranged from US$150 to US$300 per YLS in rural India and from US$150 to US$350 per YLS in urban India. Cost-effectiveness ratios are also expressed as percent of the per capita GDP (US$1,068). Even the most intensive and effective strategic package was well below 50% of the per capita GDP. In contrast to these integrated strategies, implementing only the stepwise intrapartum care upgrades—without family planning and safe abortion—was less effective and less cost-effective. The incremental cost-effectiveness ratios ranged from US$490–US$1,060 in rural India and US$200–US$990 per YLS in urban India. |
| (Graff and Amoyaw 2009) [Ghana] | Identify barriers to sustainable MVA supply | Key stakeholders | Situational assessment with interviews from key stakeholders and review of evidence | The findings of this study highlight the importance of establishing a low-cost and high-quality sustainable supply of MVA, especially for the low-volume, low- income providers. There were great accomplishments by the Government of Ghana, but otherwise there was little effort to scale-up MVA supply, which would have required an investment by donors that was not forthcoming at the time. Training activity provided by Ipas, the lead supplier of MVA equipment in Ghana, essentially ceased in 2001 with the imposition of the Mexico City Policy/Global Gag Rule. Other than free equipment obtained from trainings, MVA was available from limited private retailers in urban areas who imported equipment from outside of the country. Barriers to MVA availability in Ghana are experienced on both the supply and demand side. |
| (Gresh and Maharaj 2011) [South Africa] | To examine the acceptability of medical abortion among young people in Durban, South Africa. To investigate the potential demand for and applicability of the method among women in South Africa | Sexually active women at the University of Durban and under 30 years of age (n=20) | Qualitative in-depth interviews | Abortion services are free in public hospitals, however few people are aware of this legislation and seek costlier private providers or less safe abortions. |
| (Grossman, Grindlay et al. 2016) [Global] | Investigate public funding policies for abortion in countries with liberal or liberally interpreted laws | Public funding policies for abortion | Survey questionnaire | Among the world's female population aged 15 – 49 in countries with liberal/liberally interpreted abortion laws, 46% lived in countries with full funding for abortion (34 countries), 41% lived in countries with partial funding (25 countries), and 13% lived in countries with no funding or funding for exceptional cases only (21 countries). Thirty-one of 40 high-income countries provided full funding for abortion (n=20) or partial funding (n=11); 28 of 40 low- to middle-income countries provided full (n=14) or partial funding for abortion (n=14). Of those countries that did not provide public funding for abortion, most provided full coverage of maternity care. |
| (Guttmacher, Kapadia et al. 1998) [South Africa] | Examine the policies that have regulated accessibility of abortion and assesses their impact on reproductive health | National level policies and health outcomes | Review of policies and related evidence on health outcomes | Availability of state-funded abortions is limited. Such abortions are not available at many local clinics because of a shortage of trained providers and adequate technology. Currently, state funded terminations are only available at secondary or tertiary facilities, where the necessary resources are not as scarce. A woman can get an appointment at such a facility only after being referred by a community clinic or local day hospital. If it is economically feasible, a woman can arrange to have an abortion at a private freestanding clinic or with a private doctor, as long as these providers are registered with the state. |
| (Haas-Wilson 1996) [United States] | Estimate the impact of enforced abortion restrictions on minors' demand for abortion services between 1978-1990 | Minors who sought an abortion | Regression analysis | This paper includes estimates of the impact of the enforced abortion restrictions on minors' demand for abortions between 1978 and 1990. Using four estimation methods that account for difficult-to-measure variables, such as anti-abortion sentiment, the results suggest that parental involvement laws decrease minors' demand for abortions by 13 to 25 percent and state restrictions on Medicaid funding of abortions decrease minors' demand for abortions by 9 to 17 percent. |
| (Haas-Wilson 1997) [United States] | Determine whether Medicaid funding restrictions affect women’s reproductive health outcomes | Women of reproductive age | Regression analysis | Results indicate that abortion rates in states with Medicaid funding restrictions are 2% lower than rates in states with no such restrictions. However, when the supply of abortion providers and the demographic characteristics of the state population are taken into account, the difference is no longer statistically significant. Medicaid funding restrictions have no impact on birthrates, and the result is the same regardless of whether the empirical model takes into account provider availability, demographic characteristics and state sentiment toward women and reproductive rights. |
| (Henshaw 1995) [United States] | Provide a range of descriptive statistics on abortion access and availability in the United States | Abortion providers | Survey | Although abortion services are readily available in large urban areas to those able to pay, a 1993 survey of U.S. abortion providers shows that access to service is still problematic for many women because of barriers related to distance, gestation limits, costs and harassment. The exclusion of abortion from Medicaid coverage in most states is perhaps the most severe legislative restriction now in effect. |
| (Henshaw and Finer 2003) [United States] | Document the current status of abortion service accessibility in the United States | Abortion providers | Survey | As of December 2000, four states† of the 34 that do not fund abortions under Medicaid had legislation prohibiting private insurance from covering abortions except under an optional rider at additional cost, but these could not account for much of the difference between Medicaid-funding and non-funding states. Evidently, some of the same state characteristics that influence states to cover abortion under Medicaid influence private insurers to cover abortion. The proportion of providers that bill private insurance for their clients’ abortions is higher than average for nonhospital providers performing fewer than 30 abortions per year and for physicians’ offices. Direct billing of private insurance is most common in the Northeast (27%) and is least common in the South (5%). |
| (Hu, Bertozzi et al. 2007) [Mexico] | To conduct a cost effectiveness analysis of alternative strategies to reduce maternal mortality and morbidity in Mexico | A computer-based model that simulates the natural history of pregnancy and pregnancy-related complications in a cohort of 15-year-old women followed over their lifetime | An empirically calibrated model that simulates the natural history of pregnancy and pregnancy-related complications | Under base case assumptions, the incremental cost-effectiveness ratio associated with provision of safe abortion for all women desiring elective termination of pregnancy, but with no other changes or improvements in any dimension of safe motherhood relative to standard care, was approximately $1,400 per YLS, less than 25% of the GDP per capita. If the underlying rate of abortion in a pregnant woman is increased by 1.5, the case fatality rate due to unsafe abortion is increased to 0.002 or greater, the proportion of unsafe abortion to safe abortion is increased by 12.5%, and/or if the rate of attributable morbidity and costs of that morbidity are more than 2 times higher, the incremental cost-effectiveness ratio associated with provision of safe abortion is less than 10% of the GDP and in many cases less than 5% of the GDP (corresponding to cost-effectiveness ratios of $100 to $500 per YLS or DALY averted). |
| (Hu, Grossman et al. 2010) [Sub-Saharan Africa] | To explore the policy implications of increasing access to safe abortion in Nigeria and Ghana | Women seeking abortion | Decision analytic model | Examined hospital - based dilatation and curettage, hospital - and clinic - based manual vacuum aspiration (MVA), and medical abortion using misoprostol (MA). Assuming all modalities are equally available, clinic - based MVA is the most cost - effective option in Nigeria. If clinic - based MVA is not available, MA is the next best strategy. Conversely, in Ghana, MA is the most cost - effective strategy, followed by clinic - based MVA if MA is not available. |
| (Hyman, Baird et al. 2008) [Nepal, Viet Nam, South Africa] | To describe the experiences and lessons learned in Nepal, Viet Nam and South Africa, and make recommendations for delivering safe and effective second trimester abortions | Technical assistance provided by Ipas to the three governments | Reflections (evaluations) of the technical assistance provided | In Viet Nam, the government does not regulate patient fees, so the cost of abortion varies: In early 2008, MVA for first trimester abortion averaged around US$4–7 compared to about US$20–25 for medical abortion; D&E cost about US$80–100. The Nepal Strategic Plan specifies that the cost of second trimester abortions should be the same as first trimester abortions. Second trimester abortions cost an estimated 1,000 rupees (16 USD) for D&E or medical induction). In South Africa, all facilities are free. |
| (Jewell and Brown 2000) [United States] | This paper applies a model of fertility control to estimate the responsiveness of teenage demand for abortion to travel distance | Teenagers in Texas | Regression analysis | The results indicate that counties with higher travel costs have lower teenage abortion rates: the coefficient sizes imply that a $1.00 increase in travel cost would decrease abortions per woman by 0.86% and per pregnancy by 0.67%, other factors constant. To the degree that abortion services are available at non-licensed facilities within a county, these coefficients may overestimate the true impact of travel cost on abortion demand–as licensed providers become less available, teenage women may substitute toward having abortions at non-licensed facilities. The mean travel cost in a county that currently has abortion providers is $3.61. Suppose that abortion services were no longer available in such a county. This reduction in abortion availability increases the travel cost of acquiring an abortion to $15.48, the mean travel cost for counties without abortion providers. The increase of $11.87 in travel cost would reduce teenage abortion rates by 10.2% per woman and by 8.0% per pregnancy, translating into approximately 20 fewer abortions per year. |
| (Johnston, Gallo et al. 2007) [Uganda] | Establish the utility of the model and assess the order of magnitude of the difference in costs of care when different service delivery approaches are used | Cost data | Estimations of costs for unsafe abortions | The mean per-case cost of abortion care (in US dollars) was $45 within the setting that placed heavy restrictions on elective abortion and used a conventional approach to service delivery; $25 within the restrictive legal setting that used recommended interventions for treating complications; $34 within the legal setting that allowed elective abortion and relied on a conventional approach to service delivery; and $6 within the liberal legal setting that used recommended interventions. Using recommended technical interventions substantially reduced costs regardless of the legal setting. The greatest reduction in costs (86%) occurred from using recommended interventions within a liberal legal setting rather than using conventional interventions within a restricted setting. These findings should support policy and practice efforts to reform abortion laws and to offer accessible, safe abortion services. |
| (Johnston, Oliveras et al. 2010) [Bangladesh] | Estimate comparative costs to the health system of providing menstrual regulation and care for abortion complications | Government health facilities that provide menstrual regulation or care for abortion complications | Cost estimates | The incremental costs per case of providing menstrual regulation care in 2008 were 8-13% of those associated with treating severe abortion complications, depending on the level of care. An estimated 263,688 menstrual regulation procedures were provided at public-sector facilities in 2008, with incremental costs estimated at US$2.2 million, and 70,098 women were treated for abortion-related complications in such facilities, with incremental costs estimated at US$ 1.6 million. The provision of menstrual regulation averts unsafe abortion and associated maternal morbidity and mortality, and on a per case basis, saves scarce health system resources. Increasing access to menstrual regulation would enable more women to obtain much-needed care and health system resources to be utilized more efficiently. |
| (Jones and Weitz 2009) [United States] | Study addresses a set of laws that directly target abortion providers, make the provision of abortion more difficult and costly, and provide strong incentives for physicians not to offer abortion services. | Entire US population | Legal review | Combined with the public controversy over abortion, confusion over insurance coverage prompts many women to pay out of pocket rather than seek coverage clarification. Women who choose to pay for their abortions themselves also cite concerns about confidentiality and privacy. Finally, some abortion clinics do not accept third-party payers. Together, these factors cause three quarters of women receiving outpatient abortions to pay for the procedure with their own funds. Women with limited financial resources can find themselves in a vicious cycle: by the time they have secured the money for an abortion performed at one gestational limit, their pregnancy has advanced into the next. Studies continue to demonstrate that lack of financial support for abortion results in delays that push the procedure into the second trimester. |
| (Kahane 2000) [United States] | Estimate the effects of anti-abortion activity on the demand and supply of abortion services in 1992 | Abortion rates at the state level. | Regression analysis | Anti-abortion activities (measured as picketing with physical contact or blocking of patients) have decreased the market equilibrium abortion rate by an estimated 19 percent and raised the price of an abortion by approximately 4.3 percent. Taken together, the empirical results show that anti-abortion activities have been successful in making abortion services scarcer. |
| (Kay, Katzenellenbogen et al. 1997) [South Africa] | Analyze the medical costs incurred in treating women for incomplete abortion | Women with incomplete abortions | Cost estimates | A conservative estimate of the total cost of treating women is R18.7 million ± R3.5 million for 1994. An estimated R9.74 million ± R1.3 million of this was spent treating women with 'unsafe' incomplete abortions. |
| (Le, Connolly et al. 2015) [South Africa] | To evaluate the likely costs of unintended pregnancy in South Africa using a deterministic modeling approach | Model estimates based on contraception prevalence and failure rates among women of reproductive age. | A decision analytic model to estimate costs of unintended pregnancies | Estimated costs of miscarriages and abortion for a single year were 287 million and 282 million RAND respectively. |
| (Limacher, Daniel et al. 2006) [Canada] | Compares the costs of four options for early medical and surgical abortion in Ontario. Costs are considered from the perspectives of society, the health care system, and the patient | Abortion providers | Cost estimates | The medical options for early abortion compare favorably with the surgical options in terms of total cost to society, the health care system, and the patient. For society and the health care system, the direct costs of medical abortion are also less than those of surgical abortion, but for patients the direct costs of medical abortion are higher. Although the procedures and outcomes for early surgical abortion are essentially identical in the hospital and clinic settings, the clinic has a cost advantage from the perspectives of both society and the health care system because of its lower overhead and its greater efficiency, which is due to specialization in a single procedure. |
| (Lince-Deroche, Harries et al. 2018) [South Africa] | To estimate the costs of public-sector abortion provision in South Africa and to explore the potential for expanding access at reduced cost by changing the mix of technologies used | Public-sector abortion provision in South Africa | A budget impact analysis using public sector abortion statistics and published cost data | The public sector performed an estimated 20% of the expected total number of abortions in 2016/17; 26% and 54% of all abortions were performed illegally or in the private sector respectively. Costs were lowest in scenarios where method mix shifting occurred. Holding the proportion of abortions performed in the public-sector constant, shifting to more cost-effective service provision (more first-trimester services with more medication abortion and using the combined regimen for medical induction in the second trimester) could result in savings of $28.1 million in the public health service over the 10-year period. Expanding public sector provision through elimination of unsafe abortions would require an additional $192.5 million...South Africa can provide more safe abortions for less money in the public sector through shifting the methods provided. |
| (Medoff 2008b) [United States] | Examines if state restrictive abortion laws increase the price providers charge for supplying abortion services | State level data on number and prices of abortions | Regression analysis | The empirical results find that the enforcement of a parental notification law and a mandatory counseling law causes an increase in the price charged by abortion providers by over 13% and 9%, respectively. Based on previous estimates of the price elasticity of abortion demand, this implies that the spillover effect of a parental notification law and a mandatory counseling law is to reduce the demand for abortion, through their increase on the price of an abortion, by between 9.4% and 13.6% for a parental notification law and between 6.5% and 9.4% for a mandatory counseling law. |
| (Medoff 2015) [United States] | Examines two questions: Do restrictive state abortion laws increase the price charged by abortion providers? And, if so, Does the increase in the price charged by abortion providers have a significant negative impact on abortion demand | Abortion providers and women of reproductive age | Regression analysis | Over the period 2000-2011, state enforcement of a two-visit law and a TRAP law are associated with an increase in the real price charged by abortion providers of 19% for a two-visit law and 25% for a TRAP law. These empirical results suggest that these two restrictive state abortion laws reduce the demand for an abortion in a state by between 13% and 15% for a two-visit law and between 17% and 19% for a TRAP law. |
| (Monea and Thomas 2011) [United States] | Estimates the costs of unplanned pregnancies | Women in the U.S. of reproductive age | Cost estimates | Lower-bound, mean and upper-bound estimates of the annual cost of unintended pregnancy are, respectively, $9.6 billion, $11.3 billion and $ 12.6 billion. Corresponding estimates of the savings that would accrue to taxpayers by preventing unintended pregnancies are $4.7 billion, $5.6 billion and $6.2 billion. The mean estimate of the taxpayer cost per publicly subsidized unintended pregnancy is $9,000; the prevention of such a pregnancy would save taxpayers about half that amount. The prevention of unintended pregnancy represents an important opportunity for the public to reap substantial savings, especially given the current fiscal climate. The enactment or expansion of cost-effective policies to prevent unintended pregnancies is therefore a timely and sensible strategy. |
| (Montouchet, Trussell et al. 2013) [United Kingdom] | Estimate the direct medical costs to the National Health Service (NHS) of unintended pregnancies in 2010 and identify populations at risk for unintended pregnancies | UK population of women who had pregnancies | Costing estimates | Proportion of pregnancy outcomes stemming from unintended pregnancies and cost of each outcome to the NHS: Cost per induced abortion = £919 ($1422); cost to the NHS of induced abortions: £142.8 ($221.2) million. |
| (Murthy and Creinin 2003) [Global] | A complete review of the history and incidence of abortion, who chooses to get an abortion, who provides that service and at what cost. The cost issue is discussed using three different viewpoints: cost to the patient, cost to the provider, cost to society – mainly in the form of government expenditure and savings | Women choosing to have medical abortions | Literature review | Cost to society: For every dollar spent on abortions for poor women, > US$4 was saved in medical and social welfare costs on pregnancy-related and newborn care over the next 2 years. This is directly translated to the taxpayers, who would have to give millions of dollars to subsidize the cost of prenatal and delivery services, as well as the cost for the mothers to continue to be on medical assistance. There is additional data of this nature in the article for the UK, France, Sweden, India, and China. Main conclusion: Medical abortion, as performed currently, is an expensive process both for providers and for the patient. However, the actual cost is commonly in the same range as surgical abortion. |
| (Parmar, Leone et al. 2017) [Zambia] | Estimate the costs of providing safe abortion and PAC services at the University Teaching Hospital, Lusaka and then projected these costs to generate indicative cost estimates for Zambia | University Teaching Hospital, Lusaka | Mixed methods | The unit cost of safe abortion lies between US$37 and U$39, and for PAC after unsafe abortion between US$47 and US$56. The national estimates have wider ranges. The annual cost of safe abortion ranges from US $221,000 to US$701,000; and for PAC after unsafe abortion, from US$403,000 to US$3.5 million. Overall, the annual cost savings lie between US$66,000 and US$1.2 million, with a base estimate of US$375,000. |
| (Prada, Maddow-Zimet et al. 2013) [Colombia] | This study has multiple aims: estimate the costs incurred by health care facilities in treating complications of unsafe abortion; estimate the total annual cost to the health system of providing post-abortion care; compare the cost of treating complications of unsafe abortion with the cost of providing legal abortion services | Estimates based on a sample of facilities | Cost estimations | Cost to the National Health System. By applying the median direct cost per case to the estimated number of women receiving post-abortion care in tertiary and secondary facilities in 2012, approximately $14.4 million was spent that year on the treatment of abortion complications (not shown). This does not include indirect costs, which, as stated previously, account for around two-thirds of the cost to Colombia’s health system. Including these indirect costs, approximately $44 million was spent in 2012 on postabortion care in Colombia. |
| (Prata, Sreenivas et al. 2010) [34 sub-Saharan African countries] | To guide policy-decision makers in prioritizing the different components of safe motherhood programs in resource-scarce settings. | Different cost models for 34 different countries | Cost effectiveness models | Estimates three levels of costs – low, medium and high. The most cost-effective interventions are family planning and safe abortion, as well as anc-miso. The major contributor to variance for deaths due to unsafe abortions was cost of family planning per client and contraception prevalence. |
| (Shearer, Walker et al. 2010) [Global] | Evaluate the quality of costing studies of post-abortion care from low- and middle-income countries and to describe costs in various settings | Post-abortion care facilities across countries | Systematic review and regression analysis | Data indicate that the cost (in 2007 international dollars) of post-abortion care in Africa and Latin America is $392 and $430, respectively, per case. Differences in post-abortion care costs were associated with region, procedure, facility level, case severity, and whether the study was operations research. |
| (Sheldon and Fletcher 2017) [United Kingdom] | To provide a detailed reassessment of the relevant law and the clinical evidence that supports this assumption (that vacuum aspiration for induced abortion could be safely and legally performed by nurses and midwives) | Medically-trained abortion providers Doctors, nurses, midwives. | Reassessment of relevant law and clinical evidence | At a time of extreme budgetary restraint within the NHS, the current understanding of the law has resulted in the (relatively expensive) time of doctors being devoted to work that might safely be done by nurses and midwives. The latter are also likely to constitute a more stable workforce than junior doctors, who will move around during their career progression. Allowing nurses to provide this service thus potentially offers a more sustainable and economically efficient basis for the long-term development of excellent care. It would free up doctors to focus on those aspects of service provision where their specific expertise is needed. It might also improve the job satisfaction of nursing and midwifery staff, potentially impacting positively on sickness absence and staff retention rates. |
| (Singh 2010) [Global] | Review the scientific evidence on the consequences of unsafe abortion, highlight gaps in the evidence base, and suggest areas where future research efforts are needed. | Women who obtained unsafe abortions | Literature review | The total annual cost of providing postabortion care including overhead and capital costs in Latin America and Africa was estimated to range between $227 and $320 million in 2006 (and the average cost was $274 million). Rough estimates were made for Asia assuming that the average cost was similar to that in sub-Saharan Africa and Latin America, resulting in an estimated total annual cost of postabortion care in the developing world of just over $500 million. |
| (Thomas, Schmid et al. 2010) [United Kingdom] | To determine whether it is worth paying more for ulipristal acetate (UPA), a new method of emergency contraception, in order to avoid an additional unintended pregnancy | Women who present to NHS after having unprotected sexual intercourse | Cost-effectiveness analysis | Looking specifically at women who become pregnant following EHC (emergency hormonal contraception) in the clinical trials (HRA Pharma, data on file, June 2010) the percentage of unintended pregnancies that end in delivery is 18%, induced abortion 66% and miscarriage 16%. Applying these percentages to the average cost of a delivery (£2380), induced abortion (£672) and miscarriage (£474) gives an average cost of an unintended pregnancy of £948. |
| (Tunc 2008) [United States] | Review the vacuum aspirator's history and why, in less than a decade, electric vacuum suction became American physicians' abortion technology of choice | Americans involved in the provision/  receipt of abortion | Historical review | This article examines why, in less than a decade, electric vacuum suction became American physicians' abortion technology of choice. It focuses on factors such as political and professional feasibility (the technology was able to complement the decriminalization of abortion in the US and the interests, abilities, commitments, and personal beliefs of physicians); clinical compatibility (it met physician/patient criteria such as safety. simplicity and effectiveness); and economic viability (it was able to adapt to market factors such as production. cost, supply/demand, availability, and distribution). |
| (Vach, Bishop et al. 1998) [Vietnam] | To determine the proportion of unnecessary procedures being performed…. And estimate the costs and savings of using pregnancy testing before menstrual regulation | Women seeking menstruation regulation | Cohort study and cost estimates | Of women seeking menstrual regulation, 17% had negative pregnancy tests. If this proportion is applicable to Vietnam as a whole, some 136,000 of the estimated 800,000 menstrual regulation procedures performed each year are unnecessary. Overall, these 800 000 procedures cost the Vietnamese government about $2.2 million a year and cost women about $7 million. By providing pregnancy tests for all women seeking menstrual regulation (at a fetal cost of $720,000), the government would avoid spending $380,000 for unnecessary procedures, for a net testing cost of $340,000. Assuming costs of more than $12.00 per menstrual regulation procedure for women with complications and $8.50 for those with none, the avoidance of unnecessary procedures would save Vietnamese women an estimated total of $1.2 million. |
| (Vlassoff, Walker et al. 2009) [Global] | To estimate the health system costs of postabortion care in Africa and Latin America | Top down approach: PAC patients | Cost estimations | This study found that the health system costs of postabortion care in Africa and Latin America ranged from $159 million to $476 million per year, depending on the estimation method used. This study could not assess the cost of postabortion care in Asia or the developed world because no empirical data were available. A very approximate minimum estimate of the cost of such care in this region would be $154 million per year. Adding this to the corresponding figure for Africa and Latin America ($187 million) yields a minimum annual estimate of $341 million for postabortion care in the developing world. |
| (Vlassoff, Fetters et al. 2012) [Ethiopia] | To address the knowledge gap that exists in costing unsafe abortion in Ethiopia, estimates were derived of the cost to the health system of providing postabortion care (PAC) | 14 health facilities | Cost analysis | The average direct cost per client, across 5 types of abortion complications, was US $36.21. The annual direct cost nationally ranged from US $6.5 to US $8.9 million. Including indirect costs and satisfying all demand increased the annual national cost to US $47 million. PAC consumes a large portion of the total expenditure in reproductive health in Ethiopia. |
| (Vlassoff, Mugisha et al. 2014) [Uganda] | Presents estimates based on the research conducted in 2010 of the cost to the Ugandan health system of providing post-abortion care (PAC), filling a gap in knowledge of the cost of unsafe abortion | 39 health facilities | Cost analysis | Results show that the average annual PAC cost per client, across five types of abortion complications, was $131. The total cost of PAC nationally, including direct non-medical costs, was estimated to be $13.9 million per year. Satisfying all demand for PAC would raise the national cost to $20.8 million per year. This shows that PAC consumes a substantial portion of the total expenditure in reproductive health in Uganda. |
| (Vlassoff, Musange et al. 2015) [Rwanda] | estimate the cost to the Rwandan health-care system of providing post-abortion care (PAC) due to unsafe abortions | PAC in Rwanda | economic costing | The average annual PAC cost per client, across five types of abortion complications, was $93. The total cost of PAC nationally was estimated to be $1.7 million per year, 49% of which was expended on direct non-medical costs. Satisfying all demands for PAC would raise the national cost to $2.5 million per year. |
| (Vlassoff, Singh et al. 2016) [Ethiopia, Uganda, Rwanda and Colombia] | The objective of this study is to expand the research findings of four previous studies on the cost of post-abortion care (PAC), making use of their extensive datasets | Comparative study of costing studies from 4 countries | 4 country comparative costing study | The labor cost component varies widely: in Ethiopia and Colombia doctors spend about 30–60% more time with PAC patients than do nurses; in Uganda and Rwanda an opposite pattern is found. Labor costs range from I$42.80 in Uganda to I$301.30 in Colombia. The cost of drugs and supplies does not vary greatly, ranging from I$79 in Colombia to I$115 in Rwanda. Capital and overhead costs are substantial amounting to 52–68% of total PAC costs. Total costs per PAC case vary from I$334 in Rwanda to I$972 in Colombia. The financial burden of PAC is considerable: the expense of treating each PAC case is equivalent to around 35% of annual per capita income in Uganda, 29% in Rwanda and 11% in Colombia. Providing modern methods of contraception to women with an unmet need would cost just a fraction of the average expenditure on PAC: one year of modern contraceptive services and supplies cost only 3–12% of the average cost of treating a PAC patient. |
| (Wilder 2000) [Israel] | This study uses data from the 1974-75 Israel Fertility Survey and the 1987-88 Study of Fertility and Family Formation to examine the changing determinants of abortion among Jewish women in Israel. | Women of reproductive age in Israel | Regression analysis | Sick funds typically cover the costs of the procedure for minors and for women who have medical grounds for abortion. Abortions due to rape are funded by government welfare agencies, although full payment has been provided only since October 1991. Otherwise, the woman pays the equivalent of US$250-400, depending on the hospital, for the procedure and associated care. Abortion is widely available in Israel, and 95% of Israeli women have access to moderately priced abortion. |
| (Winikoff, Hassoun et al. 2011) [United States, France] | To examine the commercial, political, regulatory, and legislative history of the introduction of mifepristone / misoprostol in France and the United States. | Abortion providers and seekers | Review | In the United States, a large majority of women (74%) pay for their abortions with their own money or with funds they obtain from their partners, family or others. Most abortion costs are paid out-of-pocket. The insurance situation in the United States has important implications for the use and accessibility of mifepristone. On the one hand, because few women use insurance to cover the procedure, insurance companies exert little influence over practice patterns or standards of care compared to other surgical or reproductive health procedures; clinics and providers have been free to develop innovative service models for provision of the service that ultimately may have reduced the overall cost of the procedure. On the other hand, the price of the procedure has a wide range. The adjusted cost of providing medical abortion care varies significantly depending upon the practice model used (from $252 to $460 per abortion, median $351). Consequently, the method may be more or less accessible or a more or less attractive alternative to surgical abortion depending upon the practice and pricing model in place. |
| (Wu, Maru et al. 2017) [Nepal] | To review abortion care in Nepal 15 years after it was legalized | This review paper talks about abortion in relation to practitioners, women seeking abortions, national policies, etc. | Review | While the landmark 2009 Supreme Court decision established the legal framework for the government to mandate free and accessible abortion services in the public sector, there was no policy to implement safe abortion services until the passage of the Safe Abortion Service Guidelines of 2016. Under these guidelines, all government facilities should provide free abortion services. However, the provider reimbursement scheme outlined in the guidelines is less profitable for providers than it was when women paid out of pocket. It remains to be seen whether these new guidelines thus create monetary incentives that encourage providers to shift abortion provision from the public to the private sector, thereby adversely affecting access at public facilities. Despite the legal reforms, however, further improvement in protocols and infrastructure is necessary to ensure that all women truly have equal access to affordable services. Second-trimester services, for example, remain extremely limited, with many women still lacking access. |

**References**

Aantjes, C. J., A. Gilmoor, E. V. Syurina and T. L. Crankshaw (2018). "The status of provision of post abortion care services for women and girls in Eastern and Southern Africa: a systematic review." Contraception **98**(2): 77-88.

Adamczyk, A. and M. Valdimarsdóttir (2018). "Understanding Americans' abortion attitudes: The role of the local religious context." Social Science Research **71**: 129-144.

Agadjanian, V. (2002). "Is "Abortion Culture" Fading in the Former Soviet Union? Views about Abortion and Contraception in Kazakhstan." Studies in Family Planning **33**(3): 237-248.

Almond, D., L. Edlund and K. Milligan (2013). "Son Preference and the Persistence of Culture: Evidence from South and East Asian Immigrants to Canada." Population and Development Review **39**(1): 75-95.

Ananat, E. O., J. Gruber, P. B. Levine and D. Staiger (2009). "Abortion and Selection." Review of Economics and Statistics **91**(1): 124-136.

Ayanore, M. A., M. Pavlova, R. Biesma and W. Groot (2017). "Stakeholder's experiences, expectations and decision making on reproductive care: An ethnographic study of three districts in northern Ghana." PLoS One **12**(11): e0186908.

Azize-Vargas, Y. and L. A. Avilés (1997). "Abortion in Puerto Rico: The limits of colonial legality." Reproductive Health Matters **5**(9): 56-65.

Babigumira, J. B., A. Stergachis, D. L. Veenstra, J. S. Gardner, J. Ngonzi, P. Mukasa-Kivunike and L. P. Garrison (2011). "Estimating the costs of induced abortion in Uganda: a model-based analysis." BMC Public Health **11**(1): 904-904.

Baird, B. (2015). "Medical abortion in Australia: a short history." Reproductive Health Matters **23**(46): 169-176.

Baird, B. (2017). "Decriminalization and Women's Access to Abortion in Australia." Health & Human Rights: An International Journal **19**(1): 197-208.

Battistelli, M. F., S. Magnusson, M. A. Biggs and L. Freedman (2018). "Expanding the Abortion Provider Workforce: A Qualitative Study of Organizations Implementing a New California Policy." Perspectives on Sexual & Reproductive Health **50**(1): 33-39.

Benson, J., H. Gebreselassie, M. A. Manibo, K. Raisanen, H. B. Johnston, C. Mhango and B. A. Levandowski (2015). "Costs of postabortion care in public sector health facilities in Malawi: a cross-sectional survey." BMC Health Serv Res **15**: 562.

Berer, M. (2000). "Making abortions safe: a matter of good public health policy and practice." Bull World Health Organ **78**(5): 580-592.

Berer, M. (2005). "Medical abortion: issues of choice and acceptability." Reproductive Health Matters **13**(26): 25-34.

Bessett, D., K. Gorski, D. Jinadasa, M. Ostrow and M. J. Peterson (2011). "Out of Time and Out of Pocket: Experiences of Women Seeking State-Subsidized Insurance for Abortion Care in Massachusetts." Women's Health Issues **21**(3, Supplement): S21-S25.

Blanchard, K., J. L. Meadows, H. R. Gutierrez, C. P. S. Hannum, E. F. Douglas-Durham and A. J. Dennis (2017). "Mixed-methods investigation of women’s experiences with second-trimester abortion care in the Midwest and Northeast United States." Contraception **96**(6): 401-410.

Blank, R. M., C. C. George and R. A. London (1996). "State Abortion Rates: The Impact of Policies, Providers, Politics, Demographics, and Economic Environment." Journal of Health Economics **15**(5): 513-553.

Brown, R. W. and R. T. Jewell (1996). "The Impact of Provider Availability on Abortion Demand." Contemporary Economic Policy **14**(2): 95-106.

Bullard, K. A., B. L. Shaffer, K. S. Greiner, A. E. Skeith, M. I. Rodriguez and A. B. Caughey (2018). "Twenty-Week Abortion Bans on Pregnancies With a Congenital Diaphragmatic Hernia: A Cost-Effectiveness Analysis." Obstetrics & Gynecology **131**(3): 581-590.

Calkin, S. (2019). "Towards a political geography of abortion." Political Geography **69**: 22-29.

Chevrette, M. and H. A. Abenhaim (2015). "Do State-Based Policies Have an Impact on Teen Birth Rates and Teen Abortion Rates in the United States?" Journal of Pediatric and Adolescent Gynecology **28**(5): 354-361.

Coast, E., A. H. Norris, A. M. Moore and E. Freeman (2018). "Trajectories of women's abortion-related care: A conceptual framework." Social Science & Medicine **200**: 199-210.

Coles, M. S., K. K. Makino, N. L. Stanwood, A. Dozier and J. D. Klein (2010). "How Are Restrictive Abortion Statutes Associated With Unintended Teen Birth?" Journal of Adolescent Health **47**(2): 160-167.

Cook, P. J. (1999). "The Effects of Short-Term Variation in Abortion Funding on Pregnancy Outcomes." Journal of Health Economics **18**(2): 241-257.

Creinin, M. D. (2000). "Randomized comparison of efficacy, acceptability and cost of medical versus surgical abortion." Contraception **62**(3): 117-124.

Crighton, E. and M. Ebert (2002). "RU 486 and abortion practices in Europe: From legalization to access." Women & Politics **24**(3): 13-33.

Davey, C. (2005). "Sexual and Reproductive Health and Rights in the United Kingdom at ICPD+10." Reproductive Health Matters **13**(25): 81-87.

Dawson, A., D. Bateson, J. Estoesta and E. Sullivan (2016). "Towards comprehensive early abortion service delivery in high income countries: insights for improving universal access to abortion in Australia." BMC Health Services Research **16**: 612-612.

de Bruyn, M. (2003). "Safe Abortion for HIV-Positive Women with Unwanted Pregnancy: A Reproductive Right." Reproductive Health Matters **11**(22): 152-161.

Dennis, A., R. Manski and K. Blanchard (2014). "Does Medicaid Coverage Matter? A Qualitative Multi-State Study of Abortion Affordability for Low-income Women." Journal of Health Care for the Poor & Underserved **25**(4): 1571-1585.

Dennis, A., R. Manski and K. Blanchard (2015). "A Qualitative Exploration of Low-Income Women's Experiences Accessing Abortion in Massachusetts." Women's Health Issues **25**(5): 463-469.

Díaz-Olavarrieta, C., V. M. Cravioto, A. Villalobos, N. Deeb-Sossa, L. García and S. G. García (2012). "Mexico City’s Legal Abortion Program: health workers’ experiences." Revista Panamericana de Salud Publica **32**(6): 399-404.

Donohoe, M. (2005). "Increase in obstacles to abortion: the American perspective in 2004." Journal of the American Medical Women's Association **60**(1): 16-25.

Drovetta, R. I. (2015). "Safe abortion information hotlines: An effective strategy for increasing women’s access to safe abortions in Latin America." Reproductive Health Matters **23**(45): 47-57.

Duggal, R. (2004). "The political economy of abortion in India: cost and expenditure patterns." Reproductive Health Matters **12**: 130-137.

Duggal, R. and V. Ramachandran (2004). "The Abortion Assessment Project-India: Key Findings and Recommendations." Reproductive Health Matters **12**(24): 122-129.

Dzuba, I. G., B. Winikoff and M. Pena (2013). "Medical abortion: a path to safe, high-quality abortion care in Latin America and the Caribbean." Eur J Contracept Reprod Health Care **18**(6): 441-450.

Ely, G. E., T. Hales, D. L. Jackson, E. A. Bowen, E. Maguin and G. Hamilton (2017a). "A trauma-informed examination of the hardships experienced by abortion fund patients in the United States." Health Care for Women International **38**(11): 1133-1151.

Ely, G. E., T. Hales, D. L. Jackson, E. Maguin and G. Hamilton (2017b). "The undue burden of paying for abortion: An exploration of abortion fund cases." Social Work in Health Care **56**(2): 99-114.

Ely, G. E., T. W. Hales and D. L. Jackson (2018). "A cross-cultural exploration of abortion fund patients in the USA and the Republic of Ireland, Northern Ireland and the Isle of Man." Culture, Health & Sexuality **20**(5): 560-573.

Ely, G. E., T. W. Hales, D. L. Jackson, E. Maguin and G. Hamilton (2017c). "Where are They from and How Far Must They Go? Examining Location and Travel Distance in U.S. Abortion Fund Patients." International Journal of Sexual Health **29**(4): 313-324.

Erim, D. O., S. C. Resch and S. J. Goldie (2012). "Assessing health and economic outcomes of interventions to reduce pregnancy-related mortality in Nigeria." BMC Public Health **12**: 786.

Felkey, A. J. and K. M. Lybecker (2014). "Utilization of oral contraception: The impact of direct and indirect restrictions on access to abortion." The Social Science Journal **51**(1): 44-56.

Felkey, A. J. and K. M. Lybecker (2018). "Do Restrictions Beget Responsibility? The Case of U.S. Abortion Legislation." The American Economist **63**(1): 59-70.

Fischer, S., H. Royer and C. White (2018). "The Impacts of Reduced Access to Abortion and Family Planning Services on Abortions, Births, and Contraceptive Purchases." Journal of Public Economics **167**: 43-68.

Forrest, J. D. and R. Samara (1996). "Impact of publicly funded contraceptive services on unintended pregnancies and implications for Medicaid expenditures." Fam Plann Perspect **28**(5): 188-195.

Foster, D. G., R. A. Jackson, K. Cosby, T. A. Weitz, P. D. Darney and E. A. Drey (2008). "Predictors of delay in each step leading to an abortion." Contraception **77**(4): 289-293.

Foster, D. G. and K. Kimport (2013). "Who Seeks Abortions at or After 20 Weeks?" Perspectives on Sexual & Reproductive Health **45**(4): 210-218.

Franzini, L., E. Marks, P. F. Cromwell, J. Risser, L. McGill, C. Markham, B. Selwyn and C. Shapiro (2004). "Projected economic costs due to health consequences of teenagers' loss of confidentiality in obtaining reproductive health care services in Texas." Archives of Pediatrics & Adolescent Medicine **158**(12): 1140-1146.

French, V., R. Anthony, C. Souder, C. Geistkemper, E. Drey and J. Steinauer (2016). "Influence of clinician referral on Nebraska women's decision-to-abortion time." Contraception **93**(3): 236-243.

Furedi, A. (1999). "The public health implications of the 1995 'pill scare'." Hum Reprod Update **5**(6): 621-626.

Gerber Fried, M. (1997). "Abortion in the US: Barriers to access." Reproductive Health Matters **5**(9): 37-45.

Gerdts, C., S. DeZordo, J. Mishtal, J. Barr-Walker and P. A. Lohr (2016). "Experiences of women who travel to England for abortions: an exploratory pilot study." European Journal of Contraception & Reproductive Health Care **21**(5): 401-407.

Gober, P. (1997). "The role of access in explaining state abortion rates." Social Science & Medicine **44**(7): 1003-1016.

Goldie, S. J., S. Sweet, N. Carvalho, U. C. Natchu and D. Hu (2010). "Alternative strategies to reduce maternal mortality in India: a cost-effectiveness analysis." PLoS Med **7**(4): e1000264.

Graff, M. and D. A. Amoyaw (2009). "Barriers to Sustainable MVA Supply in Ghana: Challenges for the Low-Volume, Low-Income Providers." African Journal of Reproductive Health / La Revue Africaine de la Santé Reproductive **13**(4): 73-80.

Gresh, A. and P. Maharaj (2011). "A qualitative assessment of the acceptability and potential demand for medical abortion among university students in Durban, South Africa." Eur J Contracept Reprod Health Care **16**(2): 67-75.

Grossman, D., K. Grindlay and B. Burns (2016). "Public funding for abortion where broadly legal." Contraception **94**(5): 453-460.

Guttmacher, S., F. Kapadia, N. Jim Te Water and H. de Pinho (1998). "Abortion Reform in South Africa: A Case Study of the 1996 Choice on Termination of Pregnancy Act." International Family Planning Perspectives **24**(4): 191-194.

Haas-Wilson, D. (1996). "The Impact of State Abortion Restrictions on Minors' Demand for Abortions." Journal of Human Resources **31**(1): 140-158.

Haas-Wilson, D. (1997). "Women's reproductive choices: the impact of Medicaid funding restrictions." Fam Plann Perspect **29**(5): 228-233.

Henshaw, S. K. (1995). "Factors hindering access to abortion services." Family Planning Perspectives **27**(2): 54-87.

Henshaw, S. K. and L. B. Finer (2003). "The accessibility of abortion services in the United States, 2001." Perspectives on Sexual & Reproductive Health **35**(1): 16-24.

Hu, D., S. M. Bertozzi, E. Gakidou, S. Sweet and S. J. Goldie (2007). "The costs, benefits, and cost-effectiveness of interventions to reduce maternal morbidity and mortality in Mexico." PLoS One **2**(8): e750.

Hu, D., D. Grossman, C. Levin, K. Blanchard, R. Adanu and S. J. Goldie (2010). "Cost-effectiveness analysis of unsafe abortion and alternative first-trimester pregnancy termination strategies in nigeria and ghana." African Journal of Reproductive Health **14**(2): 85-103.

Hyman, A. G., T. L. Baird and I. Basnett (2008). "Establishing Second Trimester Abortion Services: Experiences in Nepal, Viet Nam and South Africa." Reproductive Health Matters **16**(31, Supplement): 135-144.

Jewell, R. T. and R. W. Brown (2000). "An economic analysis of abortion: the effect of travel cost on teenagers." The Social Science Journal **37**(1): 113-124.

Johnston, H. B., M. F. Gallo, J. Benson, H. B. Johnston, M. F. Gallo and J. Benson (2007). "Reducing the costs to health systems of unsafe abortion: a comparison of four strategies." Journal of Family Planning & Reproductive Health Care **33**(4): 250-257.

Johnston, H. B., E. Oliveras, S. Akhter and D. G. Walker (2010). "Health System Costs of Menstrual Regulation and Care For Abortion Complications in Bangladesh." International Perspectives on Sexual & Reproductive Health **36**(4): 197-201.

Jones, B. S. and T. A. Weitz (2009). "Legal barriers to second-trimester abortion provision and public health consequences." American Journal of Public Health **99**(4): 623-630.

Kahane, L. H. (2000). "Anti-abortion activities and the market for abortion services - Protest as a disincentive." American Journal of Economics and Sociology **59**(3): 463-485.

Kay, B. J., J. Katzenellenbogen, S. Fawcus and S. A. Karim (1997). "An analysis of the cost of incomplete abortion to the public health sector in South Africa - 1994." South African Medical Journal **87**(4): 442-447.

Le, H. H., M. P. Connolly, J. B. Yu, Y. Pinchevsky and P. S. Steyn (2015). "The public health and economic consequences of unintended pregnancies in South Africa." Healthcare in Low-Resource Settings **3**(1): 7.

Limacher, J. J., I. Daniel, S. Isaacksz, G. J. Payne, S. Dunn, P. C. Coyte and A. Laporte (2006). "Early Abortion in Ontario: Options and Costs." Journal of Obstetrics and Gynaecology Canada **28**(2): 142-148.

Lince-Deroche, N., J. Harries, D. Constant, C. Morroni, M. Pleaner, T. Fetters, D. Grossman, K. Blanchard and E. Sinanovic (2018). "Doing more for less: identifying opportunities to expand public sector access to safe abortion in South Africa through budget impact analysis." Contraception **97**(2): 167-176.

Medoff, M. (2015). "THE IMPACT OF STATE ABORTION POLICY ON THE PRICE OF AN ABORTION." Behavior and Social Issues **24**: 56-67.

Medoff, M. H. (2008b). "The Spillover Effects of Restrictive Abortion Laws." Gender Issues **25**(1): 1-10.

Monea, E. and A. Thomas (2011). "Unintended Pregnancy and Taxpayer Spending." Perspectives on Sexual & Reproductive Health **43**(2): 88-93.

Montouchet, C., J. Trussell, C. Montouchet and J. Trussell (2013). "Unintended pregnancies in England in 2010: costs to the National Health Service (NHS)." Contraception **87**(2): 149-153.

Murthy, A. and M. D. Creinin (2003). "Pharmacoeconomics of medical abortion: a review of cost in the United States, Europe and Asia." Expert Opin Pharmacother **4**(4): 503-513.

Parmar, D., T. Leone, E. Coast, S. F. Murray, E. Hukin and B. Vwalika (2017). "Cost of abortions in Zambia: A comparison of safe abortion and post abortion care." Global Public Health **12**(2): 236-249.

Prada, E., I. Maddow-Zimet and F. Juarez (2013). "The Cost of Postabortion Care and Legal Abortion In Colombia." International Perspectives on Sexual & Reproductive Health **39**(3): 114-123.

Prata, N., A. Sreenivas, F. Greig, J. Walsh and M. Potts (2010). "Setting priorities for safe motherhood interventions in resource-scarce settings." Health Policy **94**(1): 1-13.

Shearer, J. C., D. G. Walker and M. Vlassoff (2010). "Costs of post-abortion care in low- and middle-income countries." International Journal of Gynecology & Obstetrics **108**(2): 165-169.

Sheldon, S. and J. Fletcher (2017). "Vacuum aspiration for induced abortion could be safely and legally performed by nurses and midwives." Journal of Family Planning & Reproductive Health Care **43**(4): 260-264.

Singh, S. (2010). "Global consequences of unsafe abortion." Womens Health (Lond) **6**(6): 849-860.

Thomas, C. M., R. Schmid, S. Cameron, C. M. Thomas, R. Schmid and S. Cameron (2010). "Is it worth paying more for emergency hormonal contraception? The cost-effectiveness of ulipristal acetate versus levonorgestrel 1.5 mg." Journal of Family Planning & Reproductive Health Care **36**(4): 197-201.

Tunc, T. E. (2008). "Designs of devices: the vacuum aspirator and American abortion technology." Dynamis **28**: 353-376.

Vach, T. H., A. Bishop, V. T. Hoa, L. X. Hien, T. D. Chien and T. I. Nguyen (1998). "Potential impact of introducing pregnancy testing into menstrual regulation services in Vietnam." International Family Planning Perspectives **24**(4): 165-169.

Vlassoff, M., T. Fetters, S. Kumbi and S. Singh (2012). "The health system cost of postabortion care in Ethiopia." International Journal of Gynecology & Obstetrics **118**: S127-133.

Vlassoff, M., F. Mugisha, A. Sundaram, A. Bankole, S. Singh, L. Amanya, C. Kiggundu and F. Mirembe (2014). "The health system cost of post-abortion care in Uganda." Health Policy & Planning **29**(1): 56-66.

Vlassoff, M., S. F. Musange, I. R. Kalisa, F. Ngabo, F. Sayinzoga, S. Singh and A. Bankole (2015). "The health system cost of post-abortion care in Rwanda." Health Policy & Planning **30**(2): 223-233.

Vlassoff, M., S. Singh, O. Tsuyoshi and T. Onda (2016). "The cost of post-abortion care in developing countries: a comparative analysis of four studies." Health Policy & Planning **31**(8): 1020-1030.

Vlassoff, M., D. Walker, J. Shearer, D. Newlands and S. Singh (2009). "Estimates of health care system costs of unsafe abortion in Africa and Latin America." International Perspectives on Sexual & Reproductive Health **35**(3): 114-121.

Wilder, E. I. (2000). "Socioeconomic and Cultural Determinants of Abortion among Jewish Women in Israel." European Journal of Population / Revue Européenne de Démographie **16**(2): 133-162.

Winikoff, B., D. Hassoun and H. Bracken (2011). "Introduction and provision of medical abortion: a tale of two countries in which technology is necessary but not sufficient." Contraception **83**(4): 322-329.

Wu, W.-J., S. Maru, K. Regmi and I. Basnett (2017). "Abortion Care in Nepal, 15 Years after Legalization

Gaps in Access, Equity, and Quality." Health and Human Rights **19**(1): 221-230.
