# Supplementary material for: The macroeconomics of abortion: A scoping review and analysis of the costs and outcomes
Source: PLoS One. 2021 May 6;16(5):e0250692. doi: 10.1371/journal.pone.0250692 (PMC8101771; doi:10.1371/journal.pone.0250692)
Supplement: S2 Appendix — (DOCX) [file pone.0250692.s003.docx]

## S2 Appendix. Summary of studies reporting macroeconomics impacts (n=66)

| **Author, year [country]** | **Aim/objective(s)** | | **Population** | | **Study type** | | **Summary of main findings** | |  |
| --- | --- | --- | --- | --- | --- | --- | --- | --- | --- |
| (Ananat and Hungerman 2012) [United States] | Considers how the diffusion of oral contraception to young unmarried women affected the number and parental characteristics of children born to these women | | Children in national sample for U.S. | | Regression analysis | | Children whose births were avoided due to pill access may have had characteristics different, and along some dimensions more advantageous, from children whose births were avoided by abortion access. In the long-term, access led to negligible changes in fertility while increasing the share of children with college-educated mothers and decreasing the share with divorced mothers. The short-term effects appear to be driven by upwardly-mobile women opting out of early childbearing while the long-term effects appear to be driven by a retiming of births to later ages. Suggestive evidence indicates that pill diffusion lowered abortions. Results suggest that abortion and the pill are on average used for different purposes by different women, but on the margin some women substitute from abortion towards the pill when both are available. | |  |
| (Anandhi 2007) [India] | To contextualize the decision to abort in terms of local cultural practices, women's employment in newly emerging peri-urban informal sector, and new forms of encoding masculinities in four villages in northern Tamil Nadu where the fertility rate is rapidly declining | | Women aged between 45-65 and 25-45 who live in the study villages that are marked by high incidence of abortion and industrial estates for pharmaceutical companies | | Interviews | | Women's decision to abort is often a process of accommodating the pressures of cultural values and beliefs. Women's decision to abort seems to be linked to a vector of factors like childcare as their exclusive responsibility, marital conflicts, son preference and belief in astrology and local religion. Since women do not have control over these social conditions, their "choice" of abortion is basically to deal with these situations. The logic of family limitation through abortion as promoted by the state population policy might be influencing women's consciousness on abortion. But how women arrive at the decision to abort is not based on the received notions of family planning or birth control, but on the basis of local, social and cultural conditions and relations that define their lives. | |  |
| (Angrist and Evans 2000) [United States] | Examine effect of teen fertility and marital status on the schooling and labor market outcomes of women who were exposed to state abortion reforms as teenagers | | Women born 1949-54 | | Regression analysis | | The first-stage teen marriage effects experienced by white women did not lead to detectable schooling or labor market consequences. In contrast the teen fertility consequences of abortion reform for black women translated into large and statistically significant increases in high school graduation rates, college attendance rates, and employment rates. The increase in schooling for black women is not reflected in an increase in earnings, most likely because the effects on earnings are too far down the causal chain that began with abortion reform. | |  |
| (Averett, Rees et al. 2002)[United States] | To estimate the determinants of being sexually active and using contraceptives at last intercourse | | The National Survey of Family Growth (NSFG) Cycle V – a nationally representative survey of 10,847 women between ages 15-44 years in 1995 | | Secondary data analysis | | There is little indication that changes at a policy level of the cost of abortion impacts sexual behaviour, nor prevalence of family planning services. | |  |
| (Azarnert 2009) [Sub-Saharan Africa] | | Analyze the relationship between abortion and female education | | school enrollment rates in secondary school | | Regression analysis | | More liberal abortion policies are associated with a higher female secondary school enrollment. It is assumed in the model that easier access to abortion decreases probability of dropping out of school for a female child in the case of an occasional pregnancy. As a consequence, it enhances parental investment in human capital of their female offspring and helps to reduce the gender gap in education. | |
| (Bailey and Lindo 2017) [United States] | Chronicles changes in childbearing, provides descriptive evidence regarding trends in use, and reviews the literature linking them to changes in childbearing and women’s economic outcomes | | Women of reproductive age and children | | Literature review | | Legalized abortion led to decreases in completed childbearing—largely due to increases in childlessness— and improvements in the material living circumstances of children.... the effects of greater access to family planning and abortion services vary by age, demographic group, type of policy intervention, and context. Third, these policy changes seem to have contributed to women’s longer term economic advancement. Studies of the Pill find effects on cohabitation, age at first marriage, educational attainment, occupational choice, and the gender wage gap. Fourth, large policy changes have increased the likelihood that children are born into households with greater material resources, likely leading to improved outcomes for children. Some of the improvements in the material resources of children reflect the greater earnings capacity of both men and women, and some reflect changes in the population who select into parenthood at different times. | |  |
| (Bendavid, Avila et al. 2011) [Sub-Saharan Africa] | Investigate the association between a country's exposure to the Mexico City Policy and the odds of abortion among women of reproductive age between 1994 and 2008 | | Women of reproductive age in 20 Sub-Saharan African countries | | Regression analysis | | Women living in highly exposed countries had 2.73 times the odds of having an induced abortion after the policy's reinstatement than during the period from 1994 to 2000 or than women living in less exposed countries. After adjustments for place of residence, educational attainment, use of contraceptives and funding for family planning and reproductive health from sources other than the United States, the estimated OR dropped to 2.55. The increase in contraceptive use proceeded at a slower pace after 2002 in countries with a high level of exposure, whereas in countries with a low exposure the increase continued at the same pace. | |  |
| (Berer 2000) [Global] | This article examines the changes in policy and health service provision required to make abortions safe | | It is based on a wide-ranging review of published and unpublished sources. | | Review | | Most authors agree that treating abortion complications in sub-Saharan Africa consumes a disproportionate amount of hospital resources. In Bangladesh, up to 50% of hospital gynecology beds are reported to be taken up with abortion complications. Women tend to wait until complications become severe before seeking help, increasing both the cost and complexity of treatment. Furthermore, women attending untrained providers have been found to make more visits for care and spend more overall than women attending trained providers in the first place. A Tanzanian study estimated that the cost per day of treating abortion complications, including the costs of drugs, meals, staying costs and surgical procedures, was more than seven times the Ministry of Health's annual per capita budget. | |  |
| (Billings and Benson 2005) [Latin America & Caribbean] | This article reviews results from 10 major PAC operations research projects conducted in public sector hospitals in seven Latin American countries, completed and published between 1991 and 2002 | | All were operations research, that is, studies designed to measure the effectiveness of an intervention in achieving service delivery outcomes | | Review | | Following relatively modest interventions, the majority of eligible patients were being treated with manual vacuum aspiration (MVA), a method preferred for safety and other reasons over the method conventionally used in the region, sharp curettage (SC). A number of studies showed improvements in contraceptive counselling and services when these were integrated with clinical treatment of abortion complications, resulting in substantial increases in contraceptive acceptance. Finally, data from several studies showed that, in most settings, reorganizing services by moving treatment out of the operating theatre and reclassifying treatment as an ambulatory care procedure substantially reduced the resources used for PAC, as well as the cost and average length of women's stay in the hospital. | |  |
| (Bloom, Canning et al. 2009) [Global] | Estimate the effect of fertility on female labor force participation in a panel of countries using abortion legislation as an instrument for fertility | | Women ages 20-44 | | Regression analysis | | Removing legal restrictions on abortion significantly reduces fertility and estimate that, on average, a birth reduces a woman’s labor supply by almost 2 years during her reproductive life. Results imply that behavioral change, in the form of increased female labor supply, contributes significantly to economic growth during the demographic transition when fertility declines. | |  |
| (Bloomer and O'Dowd 2014) [Ireland] | To consider abortion tourism in Ireland, both north and south, and how the moral conservatism present in both jurisdictions has impacted on attitudes and access to abortion | | Women seeking abortion serves in restricted setting, (Ireland) | | Mixed methods | | The moral conservatism in the Republic of Ireland and Northern Ireland is apparent in the role of religious and political institutions. Both fail to acknowledge the repeated evidence of the public and professional support for abortion law reform and the evidence of the continuance of abortion tourism. Their comments on abortion demonstrate that they fail to grasp the complex set of circumstances women find themselves in when faced with a crisis pregnancy. In denying women their agency, these comments reinforce perceived traditional feminine roles as fertile, caring and inevitable mothers, in effect forcing them into ‘reproductive labor.’ | |  |
| (Bowmaker and Emerson 2013) [Europe] | Presents an alternative theory of abortion access and marriage based on the cost of search which suggests that more liberal abortion laws may actually promote young marriage | | Women of reproductive age | | Regression analysis | | A switch to a more liberal abortion law regime is associated with an increase in marriage rates for nonteenage females. the results reported in Tables 4 and 5 for the ‘twenty-somethings’ reveal a remarkably consistent story. Across all specifications, the coefficient on the liberal abortion law dummy is positive and strongly statistically significant. | |  |
| (Brown and Jewell 1996) [United States] | To estimate directly the responsiveness of abortion demand to county-level variations in travel-cost component of the full cost of abortion services | | Abortion providers in Texas | | Log-linear regressions using data that were obtained from health facilities on each abortion performed and data on the localities of these facilities | | A higher travel cost for abortion services significantly lowers county-level abortion rates. | |  |
| (Bullard, Shaffer et al. 2018) [United States] | To estimate the effect of 20-week abortion bans on maternal and consequent neonatal health outcomes and costs in the setting of fetal congenital diaphragmatic hernia | | Women in their mid-second trimester of pregnancy with a prenatal diagnosis of congenital diaphragmatic hernia | | Cohort analytic | | Twenty-week bans were associated with both poorer health outcomes from the maternal perspective and increased costs across all ranges used for the costs, probabilities, and utilities. | |  |
| (Buonanno, Drago et al. 2011) [United States and Europe] | Studies the causal impact of demographic changes, incarceration, abortion, unemployment and immigration on crime | | Crime and abortion rates | | Regression analysis | | Does not find evidence that abortion rates reduce crime rates in Europe as much as previously found for the United States. Understanding why is beyond the scope of this paper, but future research should investigate this different response of crime rates to abortion in Europe. A possible explanation is that in Europe a strong welfare state, easy access to good education and strong family ties work as risk reducing factors that weaken the link between unwanted childbearing and crime. Overall, one of the key elements explaining the drop in crime rates in the United States seems to be ineffective in Europe. | |  |
| (Coast, Norris et al. 2018) [Global] | Present a new conceptual framework for studying trajectories to obtaining abortion-related care | | Global review paper | | Review | | International institutions can shape the availability of abortion in other national and sub-national contexts, both ideologically and financially. Health system financing (e.g. free, subsidized, insurance, co-payments) affects how abortion-related care is sought and paid for. There is increased recognition of the scale and consequences of unsafe abortion, including the costs for both women and health systems, in a range of legal settings. | |  |
| (Comendant 2005) [Moldova] | Presents information on the current abortion law, policy and services in Moldova | | National-level for Moldova | | Mixed methods | | Since 1994, to reduce the number of complications, the Ministerial Order said that all abortions should be performed in hospitals by obstetrician–gynecologists. This centralization of services and their relatively higher cost has reduced the accessibility of abortion. Still the implementation of medical abortion in Moldova faces problems. There are difficulties in the organization of service delivery as most abortions are currently being provided on an inpatient basis, and the fees include payment for a hospital stay. The cost of a medical abortion, including the cost of the pills, is much higher than for vacuum aspiration. Thus, many women will not have access to this method, preferring the cheaper option. | |  |
| (Cook 1999) [United States] | To estimate the funding effects on abortion rates and birth rates | | Women who received abortions in North Carolina and data on the number of pregnancies | | State level data analysis | | In months when state funding is not available there are fewer abortions and more unacceptable births. | |  |
| (Crane and Dusenberry 2004) [Global] | To examine the effect of the Global Gag Rule on family planning organizations in countries receiving American assistance | | Family planning organizations within multiple countries | | Literature review | | Some organizations were forced to close clinics, terminate staff and cut not only family planning programs but also programs for maternal health and well-baby care, sexual health education, youth outreach, and the prevention and treatment of sexually transmitted infections and HIV/AIDS. The Gag Rule also led to the termination of all American contraceptive supply shipments to leading family planning organizations in 29 countries. | |  |
| (Dennis, Manski et al. 2014) [United States] | To answer the following questions: (1) What do women know about the cost of abortion and the availability of Medicaid coverage for abortion? (2) Where do women obtain this information? (3) What are women’s experiences paying for care? | | Low-income women aged 18 years or older who had an abortion within the past two years and who resided in one of the four study states at the time of the abortion | | Interviews | | Findings suggest that policies regarding Medicaid coverage of abortion affect the lives of women and their families in numerous ways. Restrictive coverage policies appear to force women to take measures to raise money for an abortion that may put their health and wellbeing at risk, promote short and longer-term financial instability, and increase the difficulty of implementing an abortion decision, thereby interfering with women’s reproductive life plans. Restrictive Medicaid coverage policies also appear to have ripple effects on children, partners, and parents of women seeking abortion services. | |  |
| (Donohue and Levitt 2001) [United States] | Provide evidence that legalized abortion has contributed significantly to recent crime reductions | | Crime and abortion rates | | Regression analysis | | Offers evidence that legalized abortion has contributed significantly to recent crime reductions. Crime began to fall roughly eighteen years after abortion legalization. The five states that allowed abortion in 1970 experienced declines earlier than the rest of the nation, which legalized in 1973 with Roe v. Wade. States with high abortion rates in the 1970s and 1980s experienced greater crime reductions in the 1990s. In high abortion states, only arrests of those born after abortion legalization fall relative to low abortion states. Legalized abortion appears to account for as much as 50 percent of the recent drop in crime. | |  |
| (Donohue and Levitt 2004) [United States] | This paper is an extension of the original Donohue 2001 paper following a critique by Foote 2008 | | Crime and abortion rates | | Regression analysis | | Joyce's failure to uncover a negative relationship between abortion and crime is a consequence of his decision to focus almost exclusively on one nonrepresentative six-year period during the peak of the crack epidemic. Empirical evidence indicates that the crack-cocaine epidemic hit the high-abortion early-legalizing states earlier and more severely than other states. | |  |
| (Donohue and Levitt 2008) [United States] | This paper corrects an analysis error to their original Donohue 2001 paper based on a critique by Foote 2008 | | Crime and abortion rates | | Regression analysis | | Correcting the previous mistake does not alter the sign or statistical significance of the estimates, although it does reduce their magnitude. Using a more carefully constructed measure of abortion that better links birth cohorts to abortion exposure (by using abortion data by state of residence rather than of occurrence, by adjusting for cross-state mobility, and by more precisely estimating birth years from age of arrest data), abortion legalization reduces crime through both a cohort-size and a selection effect. | |  |
| (Dragoman and Davis 2008) [United States] | Synthesis of published literature and current practices for adolescent abortion care. | | Literature on national abortion statistics and evidence for adolescents. | | Literature review | | The availability of affordable abortion services determines if a young woman can obtain an abortion at all and how quickly. Compared with adults, barriers to care may particularly affect adolescents with limited resources. Obtaining an abortion may require significant travel because most counties in the United States (more than 80%) have no abortion provider. In most states, abortion is not covered by state Medicaid programs and poor teens may have difficulty funding the cost of care. Delays experienced by adolescents lead to increased costs and risk, as later abortions are more technically difficult and expensive. | |  |
| (Duggal 2004) [India] | Examination of the political economy of abortion care in India by reviewing cost and expenditure patterns for abortion care in India | | Population of India | | Descriptive analysis of the political economy | | This review shows that it is imperative for the state to regulate the abortion economy in India, both services and the medical profession, in order to rationalize costs and assure safe abortions for women. It would make good sense to expand the base of certified and registered abortion providers to include nurses, midwives and auxiliary nurse–midwives to provide early abortion services, as it would eliminate many of the quacks. This is more easily said than done because it will involve large-scale investment in training, strong resistance from the medical profession, require strengthening of support systems in public health services and a change in the state’s perspectives on abortion. However, such an option in terms of financing would be cost-effective and simultaneously could help to increase the credibility of the public health system and rebuild women’s willingness to use public abortion services. | |  |
| (Elias, Lacetera et al. 2017) [Global] | Analysis of differences and changes in the regulation of taboo activities around the world, including abortion | | 100 countries between 1960 and 2015 | | Regression analysis | | In the case of abortion, higher income per capita is strongly associated with the adoption of formal legislation with increasingly permissive rules. Regression estimates also indicate that democratic regimes and more economic and political rights for women are associated with more permissive abortion legislation and with the legalization of non-organized forms of prostitution. In the case of abortion, the prevailing religion affects the rate at which higher income associates with more liberal legislation. The correlation is weaker in countries with a majority of Muslim citizens, although the paper estimates more liberal regulation at lower levels of income; where Catholicism is prevalent, the paper finds restrictive laws at low levels of income but a faster adoption rate of permissive rules as income increases. | |  |
| (Ely, Hales et al. 2017a) [United States] | Use a trauma-informed lens to explore abortion-related hardships in a previously understudied group | | Status as abortion seeker and inability to pay for abortion care procedure | | Descriptive, exploratory, cross- sectional analysis of administrative health care data | | Over one-third of patients report receiving some form of public assistance, which includes a range of programs such as SNAP, which stands for Supplemental Nutrition Assistance Program, formerly known as Food Stamps, Women Infants Children vouchers (food assistance for pregnant or nursing women and young children), or unemployment beneﬁts. This ﬁnding point to the broader economic hardships experienced by abortion fund patients in the United States, and is consistent with other ﬁndings suggesting that women already on public assistance are more likely to get an abortion when faced with unintended pregnancy. This is also consistent with ﬁndings indicating that public assistance recipients already experience a wide range of “mega stressors” that are difﬁcult to address. | |  |
| (Foote and Goetz 2008) [United States] | Revisits (Donohue and Levitt 2001) that linked the unexpected decline in crime during the 1990s to the legalization of abortion | | Crime and abortion rates | | Regression analysis | | Placing these results alongside those from the corrected concluding regressions and an expanded cross-state analysis, there is no compelling evidence that abortion has a selection effect on crime. | |  |
| (Foster, LaRoche et al. 2017) [Canada] | To document women's experiences obtaining abortion care in New Brunswick (NB) before and after the Regulation 84-20 amendment; identify the economic and personal costs associated with obtaining abortion care; and examine the ways in which geography, age and language-minority status condition access to care. | | New Brunswick residents who received abortion services | | Semi-structured interviews | | Although the 2015 changes to Regulation 84-20 represent an important step in aligning New Brunswick with the rest of Canada, the amendment does little to mitigate the challenges imposed by the province's refusal to fund clinic-based abortion care within or outside of the province. This study’s findings indicate that even if the elimination of the two-physician requirement were to be fully implemented, this would have only marginal impact on women's ability to access affordable and timely abortion care. | |  |
| (Foster, Biggs et al. 2018a) [United States] | To examine the association of women receiving or being denied a wanted abortion with their children’s health and well-being | | Children and mothers | | Regression analysis | | Perinatal and child health outcomes were not different between subsequent and index children, and there was no clear pattern of delayed child development. However, mixed-effects models adjusting for clustered recruitment and multiple observations per child revealed that poor maternal bonding was more common for index children compared with subsequent children. Index children lived in households with lower incomes relative to the federal poverty level than did subsequent children (101% vs 132% of federal poverty level), and were more likely to live in households without enough money to pay for basic living expenses (72% vs 55%).These findings suggest that access to abortion enables women to choose to have children at a time when they have more financial and emotional resources to devote to their children. | |  |
| (Foster, Biggs et al. 2018b) [United States] | To determine the socioeconomic consequences of receipt versus denial of abortion | | Women seeking abortion services | | Regression analysis | | In analyses that adjusted for the few baseline differences, women denied abortions who gave birth had higher odds of poverty 6 months after denial than did women who received abortions; women denied abortions were also more likely to be in poverty for 4 years after denial of abortion. Six months after denial of abortion, women were less likely to be employed full time and were more likely to receive public assistance than were women who obtained abortions, differences that remained significant for 4 years. Women denied an abortion were more likely than were women who received an abortion to experience economic hardship and insecurity lasting years. Laws that restrict access to abortion may result in worsened economic outcomes for women. | |  |
| ("Fourteenth Amendment" 2016) [United States] | To review the Whole Woman’s Health v. Hellerstedt case on abortion restrictions | | State-level and national policy/law | | Review | | By explicitly and precisely requiring future courts to consider the burdens a law imposes on abortion access together with the benefits [that law] confer[s], the Court added clarity in at least three ways. First, it translated the undue burden inquiry into a framework with which all government branches have become intimately familiar in recent decades: cost-benefit analysis. Cost-benefit analysis is certainly a far cry from the rule-like trimester framework established in Roe and subsequently rejected by Casey. But it is a standard to which courts are more accustomed, and it will thus likely be applied with greater consistency than the undue burden standard has been applied in the past. | |  |
| (Gruber, Levine et al. 1997) [United States] | Examine the impact of increased abortion availability on the average living standards of children through a selection effect | | National sample of children born immediately after abortion was legalized | | Regression analysis | | The study ﬁnds evidence of sizable positive selection: the average living circumstances of cohorts of children born immediately after abortion became legalized improved substantially relative to preceding cohorts, and relative to places where the legal status of abortion was not changing. Results suggest that the marginal children who were not born as a result of abortion legalization would have systematically been born into less favorable circumstances if the pregnancies had not been terminated: they would have been 60 percent more likely to live in a single-parent household, 50 percent more likely to live in poverty, 45 percent more likely to be in a household collecting welfare, and 40 percent more likely to die during the ﬁrst year of life. | |  |
| (Haddad, Yanow et al. 2009) [United States] | To explore provider responses to the ban in one state in order to identify important indicators for a national study | | Facilities that provided abortions in Massachusetts | | Descriptive statistics from key informant surveys | | The 2007 SCOTUS decision to uphold the Partial-Birth Abortion Act (2003) three hospitals increased their chargers for later-trimester abortion services. Medicaid-eligible people must rely on hospitals to obtain abortions at 19 weeks to receive funding under state guidelines. | |  |
| (Hodorogea and Comendant 2010)[Armenia, Azerbaijan, Belarus, Bulgaria, Croatia, Czech Republic, Estonia, Georgia, Hungary, Kazakhstan, Kyrgyzstan, Latvia, Lithuania, Republic of Moldova, Romania, Russian Federation, Slovakia, Tajikistan, Ukraine, Uzbekistan, Commonwealth of Independent States (CIS), Central Asian Republics] | To discuss progress made in various contexts toward safe and legal pregnancy termination | | Macro, national level data from the WHO and Guttmacher Institute | | Descriptive statistics | | Legal barriers have made access to abortion in Central and Eastern Europe and Central Asia more difficult. Long distances due to clinic closures and the increased cost of abortions, not covered by medical insurance or the state, are significant barriers. | |  |
| (Johnston, Oliveras et al. 2010) [Bangladesh] | Estimate comparative costs to the health system of providing menstrual regulation and care for abortion complications | | Government health facilities that provide menstrual regulation or care for abortion complications | | Cost estimates | | If all women experiencing abortion- related complications were able to access the care they needed (and that Bangladesh has committed to providing in international agreements), the total annual health system costs of providing care for abortion-related com- plications would be much higher. The Bangladesh health system could better meet the re- productive health needs of women by making menstrual regulation more accessible, further reducing recourse to unsafe abortion. For example, the health system could ensure that each facility that is meant to provide menstrual regulation services has a provider trained and equipped to offer menstrual regulation care, increase the allowed limit for menstrual regulation from 10 to 12 weeks since last menstrual period and address the socioeconomic barriers to safe menstrual regulation services. On a national level, the incremental costs of providing essential care to women with abortion-related complications amounted to roughly US$1.6 million in 2008. It is possible that these costs of treating abortion complications could be reduced and quality of care maintained or improved through shifting of care strategies. | |  |
| (Johnston, Akhter et al. 2012) [Bangladesh] | Assess incremental health system costs of service delivery for abortion-related complications in the Bangladesh public health system | | Health facilities across Bangladesh | | Interview and survey analysis | | The study findings show that despite the presence of a decentralized MR program, designed in part to reduce complications of unsafe abortion, abortion complications remain a frequent event in Bangladesh. According to the data, on average, each tertiary facility received more than 4 patients with abortion-related complications every day; this is consistent with previously generated estimates that suggest the public health system treats roughly 70,000 such patients annually. These numbers are inexcusably high. Nonetheless, it should be noted that in a 13-country analysis of hospitalizations related to abortion complications, Bangladesh – the only country in the analysis to have an established MR or abortion program – had the lowest hospitalization rate for abortion complications per 1000 women. | |  |
| (Jones and Weitz 2009) [United States] | Examine effect of set of laws on access to and quality of abortion care in the United States | | Legal review for whole of USA | | Review article | | The costs and burdens stemming from the imposition of ASC requirements have hindered or prevented physicians in some states from providing abortions. In 2004, a state law went into effect requiring that abortions after 16 weeks’ gestation be performed in ASCs or hospitals.93 Accordingly, existing abortion providers had to meet the state ASC regulations and become licensed as ASCs to continue providing abortions after 16 weeks. Compliance with the state’s ASC requirements was difficult, particularly with respect to the physical plant, and existing facilities could not meet the standards without undertaking major renovations or moving into new buildings. For example, the physical renovations alone at one facility would cost $750,000. | |  |
| (Jones and Finer 2012)[United States] | To examine the characteristics of women having abortions at 13 weeks or later | | A national sample of 9493 women obtaining abortions in 2008 | | Regression analyses. Data analyses of the 2008 Abortion Patient Survey | | One=third of abortion-care seekers relied on health insurance. Black women were twice as likely as White women to have obtained an abortion at 16+ weeks than at 13-15 weeks.  Women with family incomes 200+% of poverty were more likely than poor women to be obtaining abortions at 16+ weeks. Women using health insurance to pay were more than twice as likely to have an abortion at 16+ weeks than were women out of pocket. | |  |
| (Jones 2017) [United States] | To determine which characteristics and circumstances were associated with obtaining very early and second-trimester abortions | | 8380 non-hospital abortion patients from the 2014 Abortion Patient Survey | | Regression analyses | | 45% of patients paid for out of pocket care for abortion, Medicaid was the second most common method of payment – the overwhelming majority of people lived in one of the 15 states that use their own Medicaid funds to cover abortion.  14% of women used private insurance, 13% used financial assistance, which refers to discounts provided by clinics or subsidies available at some facilities. | |  |
| (Jones, Ingerick et al. 2018) [United States] | To determine which characteristics are associated with prior abortion | | 8380 non-hospital abortion patients from the 2014 Abortion Patient Survey | | Regression analyses | | The odds of having a prior abortion were higher for those who paid for the procedure using public or private health insurance (OR: 1.47; 95% CI: 1.29– 1.69) / received financial assistance (OR: 1.32; 95% CI: 1.15–1.52), compared to patients who paid for the abortion out of pocket. | |  |
| (Kahane 2000) [United States] | Estimate the effects of anti-abortion activity on the demand and supply of abortion services in 1992 | | Abortion rates at the state level | | Regression analysis | | With an estimated coefficient of -7.713, this means that a 1 percent increase in anti-abortion activities (as defined) leads to a decrease in the supply of abortion services (also, as defined) by approximately 0.08. This result thus supports the hypothesis set out above that anti-abortion activities have been successful in reducing the supply of abortion services. | |  |
| (Kalist 2004) [United States] | Examine whether the liberalization of state abortion laws affects female labor force participation | | Women of reproductive age | | Regression analysis | | Results indicate that abortion, by reducing unwanted pregnancies and hence fertility rates, has increased the labor force participation rates of females, especially of single black women. The data suggest that the probability a woman works (40 or more weeks a year) increases by almost 2.0 percent in states adopting legalized abortion prior to Roe v. Wade. Furthermore, abortion had a greater effect on the participation rates of black women than white women. The probability that a black woman works in the labor force increases by almost 6 percent in states adopting legalized abortion pre-Roe v. Wade. | |  |
| (Kalsi 2015) [Taiwan] | Investigate whether the ability to prenatally sex select through legal abortion | | Households that have had abortions for family planning | | Regression analysis | | Finds evidence supporting the substitution hypothesis that prenatal gender discrimination reduces postnatal discrimination for girls later in life. Once abortion is made legal, families with a strong preference for a boy at a higher birth order (or a strong distaste for a girl at a higher birth order) choose to abort the higher order female fetus. Hence, girls born at higher birth orders after the legalization of abortion are born into families with, on average, a higher preference for girls. I find results consistent with this compositional change, with abortion legalization resulting in an increase in the average rate of university attendance of higher birth order girls by about 4.5 percentage points. It is important to realize that these results do not imply that abortion legalization increased any one girl’s likelihood to attend a university but rather increased the average rate of attendance because girls who would have experienced less education, hence driving the average rate of university attendance down, were never born. | |  |
| (Levine and Staiger 2002) [United States, Romania, Russia, German Democratic Republic, Czech Republic, Slovak Republic] | Examine the impact on fertility-related behavior of changes in abortion access | | Cross country data on abortion rates and fertility | | Regression analysis | | The model predicts that legalized abortion should lead to a reduction in the likelihood of giving birth. It also predicts that if abortion access becomes relatively inexpensive (including both monetary and psychic costs), then pregnancies would rise and births would remain unchanged or may even rise as well. The paper reviews the evidence on the impact of changes in abortion policy mainly from the United States and finds support for both predictions. Eastern European countries which changed from very restrictive to liberal abortion laws experienced a large reduction in births, highlighting the insurance value. Changes from modest restrictions to abortion available upon request, however, led to no such change in births despite large increases in abortions, indicating that pregnancies rose as well. | |  |
| (Lin and Pantano 2015) [United States] | Quantify the impact of abortion legalization on the incidence of unintended births | | National-level U.S. data | | Regression analysis | | Paper finds a strong decline in the prevalence of unintended births. Moreover, it finds that this decline is mainly driven by “pro-choice” women. It then proposes an empirical strategy to recover the effect of being “unintended” on life cycle outcomes. It uses the differential timing of abortion legalization across states interacted with the mother’s religion (which facilitates or hinders legal abortion take up) to instrument for endogenous pregnancy intention. It finds that being unintended causes negative outcomes (higher crime, lower schooling, lower earnings) over the life cycle. | |  |
| (Lince-Deroche, Harries et al. 2018) [South Africa] | To estimate the costs of public-sector abortion provision in South Africa and to explore the potential for expanding access at reduced cost by changing the mix of technologies used | | Public-sector abortion provision in South Africa | | A budget impact analysis using public sector abortion statistics and published cost data | | The total estimated costs over the projected 10-year period for abortion service provision in the public sector, assuming no changes to the current share or method mix, are estimated to be $163.6 million. Total costs are lower in scenarios with the method mix adjustments. When the proportion of procedures performed in the public sector is held constant at 20% of all abortions, shifting to a more cost-effective method mix could result in savings of $28.1 million in the health service over the 10-year period. Similarly, shifting to more cost-effective methods while increasing the share of abortions provided in the public sector to 80% of total abortions could result in savings of $91.2 million in the public health service over the 10-year period. Expanding service provision in the public sector, to potentially eliminate unsafe abortions, together with shifts to more cost-effective approaches, would require an additional $192.5 million over the next 10 years at base case costs and service volume estimates. Savings through technical efficiency gains represent an opportunity to improve service quality or expand abortion access or both. Expanding public-sector provision to 80% of all abortions would require an additional $192.5 million over the next 10 years, or $19.2 million per year, even with cost savings from shifting the method mix. However, this is a small portion of the overall public health budget, which was roughly $12 billion (140.9 billion Rands) in 2014/15. | |  |
| (Lott and Whitley 2007) [United States] | The study aims to calculate the effect of abortion on crime and out-of-wedlock-births | | National-level U.S. data | | Regression analysis | | Legalizing abortion can either increase or decrease investments in children’s human capital. This article finds that abortion increases the number of out-of-wedlock births. Using data that more directly links the criminal with age when the crime was committed, not age when arrested, and fixing the assumption in previous research that no abortions took place prior to the Roe v. Wade decision in the 45 states affected by that decision, legalizing abortions increased murders by over 7%. Linear estimates indicate that legalization increased total annual victimization costs by at least $3.2 billion. | |  |
| (Malamud, Pop-Eleches et al. 2016) [Romania] | Asks if the benefit of access to better schools is larger for children who experienced better family environments because their parents had access to abortion | | Romanian administrative data | | Regression analysis | | Although paper finds that access to abortion and access to better schools each have positive impacts, it does not find evidence of significant interactions between these shocks. While these results suggest the absence of dynamic complementarities in human capital formation, survey data suggest that they may also reflect behavioral responses by students and parents. | |  |
| (Matthews, Ribar et al. 1997) [United States] | Investigate the determinants of annual abortion rates and birthrates | | State-level data | | Regression analysis | | The incidence of abortion is found to be lower in states where access to providers is reduced and state policies are restrictive. Calculations indicate that decreased access may have accounted for about one-quarter of the 5% decline in abortion rates between 1988 and 1992. In addition, birth-rates are elevated where the costs of contraception are higher because access to obstetrician-gynecologists and family planning services is reduced. Economic resources such as higher wages for men and women and generous welfare benefits are significantly and consistently related to increased birthrates; however, even a 10% cut in public assistance benefits would result in only one birth fewer for every 212 women on welfare. Economic factors showed no consistent relationship with abortion rates. | |  |
| (Medoff 1999) [United States] | To estimate the demand for abortion by teenagers | | Teenagers in the United States | | Regression analysis | | The empirical results found teenage abortion demand was inelastic with respect to price and a normal good with respect to income. Teenage abortion demand was also found to be positively related to state Medicaid funding and labor force participation. Parental involvement laws, welfare benefits, educational attainment, and state abortion attitudes are found to have no statistically significant impact on teenage abortion demand. Teenage abortion demand was found to be coincident with the business cycle. | |  |
| (Medoff 2000) [United States] | Analyze the demand for abortion by black women | | Black women in the United States | | Regression analysis | | The empirical results find that black abortion demand is price inelastic, negatively related to unemployment conditions, and positively related to the presence of state Medicaid and a college education. These results are consistent with the findings in prior studies of all childbearing women. The only two findings at odds with those of all childbearing women were that black women's abortion demands are considerably more responsive to changes in income and do not depend on marital status. | |  |
| (Medoff 2007) [United States] | Estimates abortion demand using data about price and restrictive laws or policies | | Women of reproductive age in the United States | | Regression analysis | | State Medicaid funding is found to increase the abortion demand of women of childbearing age; while the price of an abortion, parental involvement, parental consent, and parental notification laws all have a negative effect on the demand for abortions. State mandatory waiting periods have no statistically significant impact on abortion demand. The empirical results remain robust for the abortion demand of teen minors. | |  |
| (Medoff 2008a) [United States] | Estimate the impact of various restrictive abortion laws on the demand for abortion | | Women of reproductive age in the United States | | Regression analysis | | Women in the labor force or who have a higher income, have a statistically significantly greater demand for abortion. Educated women are associated with a decreased likelihood of having an unwanted pregnancy terminated, presumably due to their lower search costs of identifying and utilizing effective birth control methods. The Evangelical Christians variable has a significantly negative impact on the abortion ratio, but no statistically significant impact on the abortion rate. This result is consistent with previous research that finds that Evangelical religiosity has a strong influence on the decision not to terminate an unwanted pregnancy (in order to avoid community disapproval and social ostracism), but a much smaller role in the decision to be sexually active. | |  |
| (Medoff 2008b) [United States] | Examines if state restrictive abortion laws increase the price providers charge for supplying abortion services | | State level data on number and prices of abortions | | Regression analysis | | The empirical results find that two types of state restrictive abortion laws—parental notification and mandatory counseling—have a spillover effect on abortion demand due to the increase in the price of an abortion that results from the higher costs imposed on abortion providers as a result of complying with each restrictive abortion law. State enforcement of a parental notification law and a mandatory counseling law causes an increase in the price charged by abortion providers by over 13% and 9%, respectively. Based on previous estimates of the price elasticity of abortion demand of between -.68 and -.99, this implies that the spillover effect of the enforcement of a parental notification law and a mandatory counseling law is to reduce the demand for abortions, through their increase in the price of an abortion, by between 9.4% and 13.6% for a parental notification law and between 6.5% and 9.4% for a mandatory counseling law. | |  |
| (Medoff 2008c) [United States] | To address whether the direct (price) and indirect (restriction abortion laws) costs of abortion affect the pregnancy rates | | Cross-sectional state data pooled over 1982, 1992 and 2000 | | Cost analysis of abortions | | A 10% increase in the real price of an abortion will cause a decrease in ten pregnancy rate by over 8%. An increase in the price of obtaining an abortion induces women to engage in ex ante pregnancy avoidance methods. A 10% increase in the real price of obtaining an abortion will cause a 6.5% decrease in the pregnancy rate of women of childbearing age. | |  |
| (Meier and McFarlane 1994) [United States] | Examine whether state family planning expenditures and abortion funding for Medicaid-eligible women affect different kinds of births | | State abortion rates | | Regression analysis | | For each additional abortion funded by a state, the number of abortions increases by 0.42. In states that funded abortions for one or more of the 7 years under analysis, there were an additional 563 900 abortions, 151 900 fewer births to teen mothers, 21 300 fewer low-birthweight babies, 23 900 fewer premature births, and 232 600 fewer births with late or no prenatal care. States that did not fund abortions had 503 800 fewer abortions, 136 500 more births to teen mothers, 18 500 more low-birthweight babies, 20 800 more premature births, and 201 900 more births with late or no prenatal care. The cumulative impact of funding or not funding abortions for Medicaid-eligible women over this time period, therefore, could be substantial. | |  |
| (Myers 2017) [United States] | Provide new evidence on the relative "powers" of contraception and abortion policy in effecting the dramatic social transformations of the 1960s and 1970s | | Women of reproductive age | | Regression analysis | | Trends in sexual behavior suggest that young women’s increased access to the birth control pill fueled the sexual revolution, but neither these trends nor difference-in-difference estimates support the view that this also led to substantial changes in family formation. Rather, the estimates robustly suggest that it was liberalized access to abortion that allowed large numbers of women to delay marriage and motherhood. Relative to the adjusted predictions, these are very large effects: liberalized abortion policy predicts a 34 percent decline in motherhood, a 20 percent decline in marriage, and a 63 percent decline in shotgun marriages prior to age 19. As an additional means of gauging the magnitude of these estimates, I use the regression coefficients to predict outcomes for the 1940 and 1958 cohorts had abortion reforms and legalization not occurred. This exercise suggests that the liberalization of abortion policy explains about 80 percent of the decline in the probability of birth and 25 percent of the decline in the probability of marriage prior to age 19 observed between these birth cohorts. | |  |
| (Oreffice 2007) [United States] | Estimates the impact of abortion legalization on spouses’ labor supplies to test whether legalization increased women’s household bargaining power | | Married couples | | Regression analysis | | Abortion legalization significantly decreased the labor supply of married women in their fertile age by 83 annual hours and significantly increased their husbands’ labor supply by 34 annual hours. In addition, the theory provides a number of other predictions. First, abortion legalization should have no effect on couples with strongly held religious beliefs against abortion. Second, abortion legalization should have little impact on couples who regularly use contraceptives. Third, to the extent that households from the upper part of the income distribution would have been able to obtain abortions more readily even when they were illegal, the effects of legalization should be less evident. The empirical evidence is consistent with these predictions. | |  |
| (Parmar, Leone et al. 2017) [Zambia] | Estimate the costs of providing safe abortion and PAC services at the University Teaching Hospital, Lusaka and then projected these costs to generate indicative cost estimates for Zambia | | University Teaching Hospital, Lusaka | | Mixed methods | | It costs the Zambian public health system 2.5 times more to provide PAC for unsafe abortions than to provide safe abortion. If women requiring PAC following unsafe abortion instead had a safe abortion, Zambia’s public health system would incur a cost saving of approximately US$375,000 per year. The projected costs for PAC for unsafe abortion in 2030 alone could be nearly US$11million if the number of abortions, contraceptive prevalence and health budget were to remain constant. The overall financial burden of PAC on the health expenditure budget could potentially increase to 0.9% (currently 0.2%). The annual cost of safe abortion ranges from US $221,000 to US$701,000; and for PAC after unsafe abortion, from US$403,000 to US$3.5 million. Overall, the annual cost savings lie between US$66,000 and US$1.2 million, with a base estimate of US$375,000. | |  |
| (Pop-Eleches 2006) [Romania] | Examines educational and labor outcomes of children affected by a ban on abortions | | All children born between January and October 1967 | | Regression analysis | | On average, children born after abortion became illegal display better educational and labor market achievements, and this outcome can be explained by a change in the composition of families having children: urban, educated women working in good jobs were more likely to have abortions prior to the policy change, so a higher proportion of children were born into urban, educated households. Moreover, the analysis shows that after I control for this type of compositional changes, the children born after the abortion ban had significantly worse schooling and labor market outcomes. I interpret this result as evidence of the existence of a negative unwantedness effect. | |  |
| (Shah, Åhman et al. 2014) [Global] | To review the evidence on abortion laws and policies, and trends in the incidence of safe and unsafe abortion and in mortality due to unsafe abortion | | Abortion seekers, countries with abortion policies | | Review | | Globally, the number of induced abortions (safe and unsafe) per 1000 women aged 15–44 years declined from 35 in 1995 to 28 in 2008. The number of deaths due to unsafe abortion declined from 69,000 in 1990 to 47,000 in 2008, as safe and effective methods of abortion, including manual vacuum aspiration and medical abortion, became more widely available. During the same period, there was a slight increase in the number of countries where abortion is permitted on request, and 70 countries made grounds for abortion more liberal. Since ICPD, the decline in unsafe abortion was slower than that in safe abortion, and unsafe-abortion-related mortality continued to be a problem. Nearly all unsafe abortions and mortality occur in developing countries. | |  |
| (Tunc 2008) [United States] | Review the vacuum aspirator's history and why, in less than a decade, electric vacuum suction became American physicians' abortion technology of choice | | Americans involved in the provision/  receipt of abortion | | Historical review | | The electric vacuum suction technique triumphed because it was politically and professionally feasible (Le., it was able to complement the decriminalization of abortion in the US, as well as the interests, abilities, commitments, and personal beliefs of physicians); clinically compatible (Le., it met physician/patient criteria such as safety, simplicity, and effectiveness); and economically viable (Le., able to adapt to market factors such as production, cost, supply/demand, availability, and distribution). The entry of the electrical vacuum aspiration machine into the medical mainstream was clearly facilitated by the fact that abortion was being decriminalized on a state-by-state basis just as physicians such as Margolis and Nathanson were discovering the apparatus. | |  |
| (Vlassoff, Walker et al. 2009) [Global] | To estimate the health system costs of postabortion care in Africa and Latin America | | Top down approach: PAC patients | | Cost estimations | | This study found that the health system costs of postabortion care in Africa and Latin America ranged from $159 million to $476 million per year, depending on the estimation method used. The average estimates from the two approaches largely coincide: $280 million using the top-down approach, and $274 million using the bottom-up approach (averaging actual and standard practice estimates). These sums are considerable and impose an added burden on the already overstretched health resources of developing countries. A new United Nations estimate of maternal and newborn health expenditures places the cost of unsafe abortion in context: Obstetric complications cost health care systems in Africa and Latin America around $490 million annually. | |  |
| (Whitaker 2011) [United States] | Examines whether the legalization of abortion changed high school graduation rates among the children selected into birth | | High school graduation rates | | Regression analysis | | Unless women in all socio-economic circumstances sought abortions to the same extent, increased use of abortion must have changed the distribution of child development inputs. Higher abortion ratios are associated with higher graduation rates for black males, but not other demographic groups. Regression results indicate abortion ratios are linked with the fertility differences between ethnicities, which suggests this is a channel of influence. Overall, the relationship between abortion exposure and educational attainment is small. There is evidence for positive selection due to legalized abortion but among only one subpopulation, black males. The results seem to point to negative selection in aggregate. As with all social phenomena, abortion availability interacted with evolving economic, cultural, and legal institutions. It was certainly part of a bundle of changing sexual practices and attitudes toward family life. It appears that abortion was used disproportionately by black women in difficult circumstances, and therefore fewer of the women’s potential sons became high school dropouts. However, more economically advantaged white women seem to have lowered their fertility even more, possibly in response to rising opportunity costs. This reduced the share of children raised with the child development inputs necessary to complete high school. | |  |
| (Wu, Maru et al. 2017) [Nepal] | To review abortion care in Nepal 15 years after it was legalized | | This review paper talks about abortion in relation to practitioners, women seeking abortions, national policies, etc. | | Review | | While the landmark 2009 Supreme Court decision established the legal framework for the government to mandate free and accessible abortion services in the public sector, there was no policy to implement safe abortion services until the passage of the Safe Abortion Service Guidelines of 2016. Under these guidelines, all government facilities should provide free abortion services. However, the provider reimbursement scheme outlined in the guidelines is less profitable for providers than it was when women paid out of pocket. It remains to be seen whether these new guidelines thus create monetary incentives that encourage providers to shift abortion provision from the public to the private sector, thereby adversely affecting access at public facilities. Despite the legal reforms, however, further improvement in protocols and infrastructure is necessary to ensure that all women truly have equal access to affordable services. Second-trimester services, for example, remain extremely limited, with many women still lacking access. | |  |

**References**

"Fourteenth Amendment" (2016). "Fourteenth Amendment - Due Process Clause - Undue Burden - Whole Woman's Health v. Hellerstedt." Harvard Law Review **130**(1): 397.

Ananat, E. O. and D. M. Hungerman (2012). "THE POWER OF THE PILL FOR THE NEXT GENERATION: ORAL CONTRACEPTION'S EFFECTS ON FERTILITY, ABORTION, AND MATERNAL AND CHILD CHARACTERISTICS." The Review of Economics and Statistics **94**(1): 37-51.

Angrist, J. D. and W. N. Evans (2000). Schooling and labor market consequences of the 1970 state abortion reforms. Research in Labor Economics, Emerald Group Publishing Limited. **18:** 75-113.

Averett, S. L., D. I. Rees and L. M. Argys (2002). "The impact of government policies and neighborhood characteristics on teenage sexual activity and contraceptive use." American Journal of Public Health **92**(11): 1773-1778.

Azarnert, L. V. (2009). "ABORTION AND HUMAN CAPITAL ACCUMULATION: A CONTRIBUTION TO THE UNDERSTANDING OF THE GENDER GAP IN EDUCATION." Scottish Journal of Political Economy **56**(5): 559-579.

Bailey, M. J. and J. M. Lindo (2017). Access and Use of Contraception and Its Effects on Women's Outcomes in the U.S, National Bureau of Economic Research, Inc, NBER Working Papers: 23465.

Bendavid, E., P. Avila and G. Miller (2011). "United States aid policy and induced abortion in sub-Saharan Africa." Bulletin of the World Health Organization **89**(12): 873-880C.

Berer, M. (2000). "Making abortions safe: a matter of good public health policy and practice." Bull World Health Organ **78**(5): 580-592.

Billings, D. L. and J. Benson (2005). "Postabortion care in Latin America: policy and service recommendations from a decade of operations research." Health Policy Plan **20**(3): 158-166.

Bloom, D. E., D. Canning, G. Fink and J. E. Finlay (2009). "Fertility, Female Labor Force Participation, and the Demographic Dividend." Journal of Economic Growth **14**(2): 79-101.

Bloomer, F. and K. O'Dowd (2014). "Restricted access to abortion in the Republic of Ireland and Northern Ireland: exploring abortion tourism and barriers to legal reform." Culture, Health & Sexuality **16**(4): 366-380.

Bowmaker, S. W. and P. M. Emerson (2013). "Still Waiting for Mister Right? Asymmetric Information, Abortion Laws and the Timing of Marriage." Applied Economics **45**(22-24): 3151-3169.

Brown, R. W. and R. T. Jewell (1996). "The Impact of Provider Availability on Abortion Demand." Contemporary Economic Policy **14**(2): 95-106.

Bullard, K. A., B. L. Shaffer, K. S. Greiner, A. E. Skeith, M. I. Rodriguez and A. B. Caughey (2018). "Twenty-Week Abortion Bans on Pregnancies With a Congenital Diaphragmatic Hernia: A Cost-Effectiveness Analysis." Obstetrics & Gynecology **131**(3): 581-590.

Buonanno, P., F. Drago, R. Galbiati and G. Zanella (2011). "Crime in Europe and the United States: Dissecting the 'Reversal of Misfortunes'." Economic Policy(67): 347.

Coast, E., A. H. Norris, A. M. Moore and E. Freeman (2018). "Trajectories of women's abortion-related care: A conceptual framework." Social Science & Medicine **200**: 199-210.

Comendant, R. (2005). "A project to improve the quality of abortion services in Moldova." Reproductive Health Matters **13**(26): 93-100.

Cook, P. J. (1999). "The Effects of Short-Term Variation in Abortion Funding on Pregnancy Outcomes." Journal of Health Economics **18**(2): 241-257.

Crane, B. B. and J. Dusenberry (2004). "Power and Politics in International Funding for Reproductive Health: the US Global Gag Rule." Reproductive Health Matters **12**(24): 128-137.

Dennis, A., R. Manski and K. Blanchard (2014). "Does Medicaid Coverage Matter? A Qualitative Multi-State Study of Abortion Affordability for Low-income Women." Journal of Health Care for the Poor & Underserved **25**(4): 1571-1585.

Donohue, J. J., III and S. D. Levitt (2001). "The Impact of Legalized Abortion on Crime." Quarterly Journal of Economics **116**(2): 379-420.

Donohue, J. J. and S. D. Levitt (2004). "Further Evidence That Legalized Abortion Lowered Crime: A Reply to Joyce." The Journal of Human Resources **39**(1): 29-49.

Donohue, J. J. and S. D. Levitt (2008). "Measurement Error, Legalized Abortion, and the Decline in Crime: A Response to Foote and Goetz." The Quarterly Journal of Economics **123**(1): 425-440.

Dragoman, M. and A. Davis (2008). "Abortion care for adolescents." Clinical Obstetrics & Gynecology **51**(2): 281-289.

Duggal, R. (2004). "The political economy of abortion in India: cost and expenditure patterns." Reproductive Health Matters **12**: 130-137.

Elias, J. J., N. Lacetera, M. Macis and P. Salardi (2017). "Economic Development and the Regulation of Morally Contentious Activities." American Economic Review **107**(5): 76-80.

Ely, G. E., T. Hales, D. L. Jackson, E. A. Bowen, E. Maguin and G. Hamilton (2017a). "A trauma-informed examination of the hardships experienced by abortion fund patients in the United States." Health Care for Women International **38**(11): 1133-1151.

Foote, C. L. and C. F. Goetz (2008). "The Impact of Legalized Abortion on Crime: Comment." Quarterly Journal of Economics **123**(1): 407-423.

Foster, A. M., K. J. LaRoche, J. El-Haddad, L. DeGroot and I. M. El-Mowafi (2017). ""If I ever did have a daughter, I wouldn't raise her in New Brunswick:" exploring women's experiences obtaining abortion care before and after policy reform." Contraception **95**(5): 477-484.

Foster, D. G., M. A. Biggs, S. Raifman, J. Gipson, K. Kimport and C. H. Rocca (2018a). "Comparison of Health, Development, Maternal Bonding, and Poverty Among Children Born After Denial of Abortion vs After Pregnancies Subsequent to an AbortionHealth, Development, Maternal Bonding, and Poverty Among Children Born After Denial of AbortionHealth, Development, Maternal Bonding, and Poverty Among Children Born After Denial of Abortion." JAMA Pediatrics **172**(11): 1053-1060.

Foster, D. G., M. A. Biggs, L. Ralph, C. Gerdts, S. Roberts and M. M. Glymour (2018b). "Socioeconomic Outcomes of Women Who Receive and Women Who Are Denied Wanted Abortions in the United States." American Journal of Public Health **108**(3): 407-413.

Gruber, J., P. Levine and D. Staiger (1997). Abortion Legalization and Child Living Circumstances: Who is the 'Marginal Child?', National Bureau of Economic Research, Inc, NBER Working Papers: 6034.

Haddad, L., S. Yanow, L. Delli-Bovi, K. Cosby and T. A. Weitz (2009). "Changes in abortion provider practices in response to the Partial-Birth Abortion Ban Act of 2003." Contraception **79**(5): 379-384.

Hodorogea, S. and R. Comendant (2010). "Prevention of unsafe abortion in countries of Central Eastern Europe and Central Asia." International Journal of Gynecology & Obstetrics **110**: S34-S37.

Johnston, H. B., S. Akhter and E. Oliveras (2012). "Quality and efficiency of care for complications of unsafe abortion: a case study from Bangladesh." International Journal of Gynecology & Obstetrics **118**: S141-147.

Johnston, H. B., E. Oliveras, S. Akhter and D. G. Walker (2010). "Health System Costs of Menstrual Regulation and Care For Abortion Complications in Bangladesh." International Perspectives on Sexual & Reproductive Health **36**(4): 197-201.

Jones, B. S. and T. A. Weitz (2009). "Legal barriers to second-trimester abortion provision and public health consequences." American Journal of Public Health **99**(4): 623-630.

Jones, R. K. (2017). "Are Uncertain Fertility Intentions a Temporary or Long-term Outlook? Findings from a Panel Study." Women's Health Issues **27**(1): 21-28.

Jones, R. K. and L. B. Finer (2012). "Who has second-trimester abortions in the United States?" Contraception **85**(6): 544-551.

Jones, R. K., M. Ingerick and J. Jerman (2018). "Differences in Abortion Service Delivery in Hostile, Middle-ground, and Supportive States in 2014." Women's Health Issues **28**(3): 212-218.

Kahane, L. H. (2000). "Anti-abortion activities and the market for abortion services - Protest as a disincentive." American Journal of Economics and Sociology **59**(3): 463-485.

Kalist, D. E. (2004). "Abortion and Female Labor Force Participation: Evidence Prior to Roe v. Wade." Journal of Labor Research **25**(3): 503-514.

Kalsi, P. (2015). "Abortion Legalization, Sex Selection, and Female University Enrollment in Taiwan." Economic Development and Cultural Change **64**(1): 163-185.

Levine, P. B. and D. Staiger (2002). Abortion as Insurance, National Bureau of Economic Research, Inc, NBER Working Papers: 8813.

Lin, W. and J. Pantano (2015). "The Unintended: Negative Outcomes over the Life Cycle." Journal of Population Economics **28**(2): 479-508.

Lince-Deroche, N., J. Harries, D. Constant, C. Morroni, M. Pleaner, T. Fetters, D. Grossman, K. Blanchard and E. Sinanovic (2018). "Doing more for less: identifying opportunities to expand public sector access to safe abortion in South Africa through budget impact analysis." Contraception **97**(2): 167-176.

Lott, J. R., Jr. and J. Whitley (2007). "Abortion and Crime: Unwanted Children and Out-of-Wedlock Births." Economic Inquiry **45**(2): 304-324.

Malamud, O., C. Pop-Eleches and M. Urquiola (2016). Interactions Between Family and School Environments: Evidence on Dynamic Complementarities?, National Bureau of Economic Research, Inc, NBER Working Papers: 22112.

Matthews, S., D. Ribar and M. Wilhelm (1997). "The effects of economic conditions and access to reproductive health services on state abortion rates and birthrates." Family Planning Perspectives **29**(2): 52-60.

Medoff, M. H. (1999). "An Estimate of Teenage Abortion Demand." Journal of Socio-Economics **28**(2): 175-184.

Medoff, M. H. (2000). "Black Abortion Demand." Review of Black Political Economy **28**(1): 29-36.

Medoff, M. H. (2007). "Price, Restrictions and Abortion Demand." Journal of Family and Economic Issues **28**(4): 583-599.

Medoff, M. H. (2008a). "The response of abortion demand to changes in abortion costs." Social Indicators Research **87**(2): 329-346.

Medoff, M. H. (2008b). "The Spillover Effects of Restrictive Abortion Laws." Gender Issues **25**(1): 1-10.

Medoff, M. H. (2008c). "Abortion costs, sexual behavior, and pregnancy rates." Social Science Journal **45**(1): 156-172.

Meier, K. J. and D. R. McFarlane (1994). "State family planning and abortion expenditures: Their effect on public health." American Journal of Public Health **84**(9): 1468-1472.

Myers, C. K. (2017). "The Power of Abortion Policy: Reexamining the Effects of Young Women's Access to Reproductive Control." Journal of Political Economy **125**(6): 2178-2224.

Oreffice, S. (2007). "Did the Legalization of Abortion Increase Women's Household Bargaining Power? Evidence from Labor Supply." Review of Economics of the Household **5**(2): 181-207.

Parmar, D., T. Leone, E. Coast, S. F. Murray, E. Hukin and B. Vwalika (2017). "Cost of abortions in Zambia: A comparison of safe abortion and post abortion care." Global Public Health **12**(2): 236-249.

Pop-Eleches, C. (2006). "The Impact of an Abortion Ban on Socioeconomic Outcomes of Children: Evidence from Romania." Journal of Political Economy **114**(4): 744-773.

Shah, I. H., E. Åhman and N. Ortayli (2014). "Access to safe abortion: progress and challenges since the 1994 International Conference on Population and Development (ICPD)." Contraception **90**(6, Supplement): S39-S48.

Tunc, T. E. (2008). "Designs of devices: the vacuum aspirator and American abortion technology." Dynamis **28**: 353-376.

Vlassoff, M., D. Walker, J. Shearer, D. Newlands and S. Singh (2009). "Estimates of health care system costs of unsafe abortion in Africa and Latin America." International Perspectives on Sexual & Reproductive Health **35**(3): 114-121.

Whitaker, S. (2011). "The Impact of Legalized Abortion on High School Graduation through Selection and Composition." Economics of Education Review **30**(2): 228-246.

Wu, W.-J., S. Maru, K. Regmi and I. Basnett (2017). "Abortion Care in Nepal, 15 Years after Legalization

Gaps in Access, Equity, and Quality." Health and Human Rights **19**(1): 221-230.
