# Supplementary material for: The macroeconomics of abortion: A scoping review and analysis of the costs and outcomes
Source: PLoS One. 2021 May 6;16(5):e0250692. doi: 10.1371/journal.pone.0250692 (PMC8101771; doi:10.1371/journal.pone.0250692)
Supplement: S3 Appendix — (DOCX) [file pone.0250692.s004.docx]

## S3 Appendix. Summary of studies reporting macroeconomics value/benefit (n=26)

| **Author, year [country]** | **Aim/objective(s)** | **Population** | **Study type** | **Summary of main findings** |
| --- | --- | --- | --- | --- |
| (Almond, Edlund et al. 2013) [Canada] | The study of South and East Asian immigrants in Canada, a rich OECD country, can potentially cast light on the role of culture in sex selection | Census data from Canada | Descriptive statistics | High and rising sex ratios lead one to ask what causes parents to prefer sons over daughters. One strand of argument emphasizes socioeconomic and institutional factors. Absent couples’ ability to save or to rely on national pensions, the poor count heavily on children for old-age support, a task that under patriarchal norms falls on sons. In India, high and rising dowry payments are argued to place families with daughters at a disadvantage, and it has also been argued that families depend on males for physical protection. In both India and China, however, sex ratios are highest in the richest areas, and for India a strong education gradient is evident, with better-educated parents favoring sons more extensively. These observations cast doubt on sex selection being the result of economic necessity alone. |
| (Ananat, Gruber et al. 2009) [United States] | Provide a framework for understanding selection mechanisms and use that framework to address inconsistent past methodological approaches and provide evidence on the long-run impact on cohort characteristics | Individuals born in the United States and observed in the 2000 Census at ages 21 to 35 in that year (born between 1965 and 1979) | Regression analysis | The results on education are perhaps most striking, with a large negative effect on the percentage of the cohort that did not graduate from college (column 5), indicating that abortion legalization shifted the distribution of education upward. But these results provide no evidence of convergence; coefficients on repeal 1974–1975 and repeal 1976 1979 are generally of the same sign and larger than the coefficient on repeal 1971–1973. This lack of convergence supports the possibility that continued growth in both the pregnancy and abortion rates in early legalization states caused greater positive selection in the composition of births in those states, even after abortion was legalized nationally and birth rates converged. |
| (Belton and Whittaker 2007) [Thailand] | To examine the needs for reproductive services of Burmese migrant workers along the border with Thailand. | Women experiencing any type of early pregnancy loss; traditional midwife trainers. | Cross-sectional descriptive | Women’s right to work for equal pay, right to work during a pregnancy or any notions of maternity leave is non-existent for Burmese workers in Thailand. As most women interviewed did not have a work permit, they were a particularly vulnerable population. |
| (Benson, Okoh et al. 2012) [Nigeria] | To add more recent cost estimates of PAC in Nigeria to the existing body of literature | 79 PAC providing  public hospitals | Cost analysis | The World Bank calculates a per capita health expenditure cost of US $69, with more than one-third of total health expenditures borne by the public sector in Nigeria. Just 1 PAC case over the 3 states consumes an estimated US $79, which is illustrative of the stress that current practices place on public health system budgets and the hidden effect on competing obstetric and gynecologic needs that may go unmet. |
| (Benson, Gebreselassie et al. 2015) [Malawi] | This study estimates current health system costs of treating unsafe abortion complications and compares these findings with newly-projected costs for providing safe abortion in Malawi | Malawi health system costs | Estimation study based on survey and costing data | Treating 18,600 women for PAC in public health facilities annually represents a significant and preventable burden on the public health system. The median cost of treating one PAC case in public facilities, $40, is markedly higher than the 2011 per capita spending by the Malawi government on health, $23. A liberalized abortion law and access to safe abortion in public health facilities yielded a 20-30% decrease in current PAC costs. This drop would occur even though a high-quality first-trimester legal abortion with MVA (with appropriate use of supplies and pain management drugs) costs somewhat more than the current treatment of a simple PAC case. |
| (Bullard, Shaffer et al. 2018) [United States] | To estimate the effect of 20-week abortion bans on maternal and consequent neonatal health outcomes and costs in the setting of fetal congenital diaphragmatic hernia | Women in their mid-second trimester of pregnancy with a prenatal diagnosis of congenital diaphragmatic hernia | Cohort analytic | Whereas the model was conducted from a societal standpoint, the payers for much of these costs would be insurers and the most prominent payer would be Medicaid, because that is the largest single payer for neonatal and maternal care. |
| (Cheng, Zhou et al. 2012) [China] | To investigate providers' knowledge and attitudes about medical abortion | Abortion service providers | Survey with multistage stratified cluster sampling design | Even though SA is generally available in China under medically safe conditions, MA provides a low-cost, very low-risk alternative with other advantages that include avoidance of surgery and anesthesia. |
| (Comendant 2005) [Moldova] | Presents information on the current abortion law, policy and services in Moldova | National-level for Moldova | Mixed methods | Providers are pleased with the MVA services which, they say, are not only safe and effective but also save time and costs. Women who have MVA are also pleased: ongoing monitoring during 2003–04 at the MVA Centre found that more than 90% were very satisfied with their care and would recommend it to others needing an abortion. |
| (Elias, Lacetera et al. 2017) [Global] | Analysis of differences and changes in the regulation of taboo activities around the world, including abortion | 100 countries between 1960 and 2015 | Regression analysis | The main finding of a positive association between income and liberal legislation for abortion and prostitution is consistent with the idea that cost-benefit considerations affect attitudes toward repugnant transactions. Economic development could affect the regulation of morally disputed transactions through three channels: direct effects on efficiency and repugnance; and an indirect effect through the change in the individuals’ relative valuation of the two policy options (legalization versus prohibition) due for example to income effects. |
| (Erim, Resch et al. 2012) [Nigeria] | Synthesize the best available data, adapt a model of pregnancy and pregnancy-related morbidity and mortality to the Nigerian context, and conduct national and regional analyses that quantify the payoffs from investing in safe pregnancy and childbirth | National level estimates | Mixed methods | Paper provides robust insight into the importance of investing in all three domains of family planning, safe abortion and intrapartum care – any approach that focuses on only one of these, to the exclusion of the other, will be less effective and cost-effective. |
| (Fletcher 2000) [Ireland] | Tease out what might be gained or lost from the categorization of abortion as a service regulated by European Community law | Abortion law | Feminist legal analysis | This paper addresses the implications of categorizing abortion as a supranational economic service for feminist legal strategy. The advantages of categorizing abortion as a service to which women have access as consumers are that it legitimates abortion and it provides a new strategy for making abortion claims. The disadvantages are that a woman's legal interest in abortion is based on her capacity to buy the service, fetal life is rendered devoid of value, and the service supplier has as much say about the abortion transaction as the woman consumer. If feminist legal strategy is to successfully use the legal construction of abortion as an economic service, it must work to minimize such negative implications. |
| (Foster, Biggs et al. 2018a) [United States] | To examine the association of women receiving or being denied a wanted abortion with their children’s health and well-being | Children and mothers | Regression analysis | The study included data on index children for a mean of 3.4 years and subsequent children for a mean of 2.2 years; outcomes such as physical growth or school performance may not be apparent in that time frame. Children born beyond 5 years after the abortion may have outcomes that further diverge from those of index children, because the woman may have more time to establish the life circumstances in which she desires to parent, such as stable relationships, completed education, and/or secure financial footing. Being able to delay childbearing even for a few years and thus have a child at a time that the woman feels is better may result in closer relationships between mother and child and children raised in better economic circumstances. |
| (Foster, Biggs et al. 2018b) [United States] | To determine the socioeconomic consequences of receipt versus denial of abortion | Women seeking abortion services | Regression analysis | Differences over time in employment, poverty, and receipt of public assistance suggest that public assistance programs served an important role in mitigating the loss of full-time employment for women denied an abortion. However, public assistance was not sufficient to support the increase in household size resulting from a new baby, and did not keep households of women denied an abortion from living in poverty. Differences in economic outcomes gradually converged over the 5 years. At the time of seeking an abortion, more than a quarter of all women in the study were living in a household as the only adult with children, and this increased significantly for women who were denied an abortion, indicating that the burden of raising a child often falls to women alone rather than to couples or an extended family. |
| (Haas-Wilson 1996) [United States] | Estimate the impact of enforced abortion restrictions on minors' demand for abortion services between 1978-1990 | Minors who sought an abortion | Regression analysis | Abortions appear to be a normal good. A 1 percent increase in per capita income results in a 1.35 to 2.07 percent increase in minors' demand for abortions. Labor force participation is associated with higher abortion rates. A 1 percent increase in the labor force participation rate of women results in a 0.56 to 1.35 percent increase in minors' demand for abortions. Education is associated with lower abortion rates. A 1 percent increase in the percentage of women who are high school graduates results in a 0.48 to 1.19 percent decrease in minors' demand for abortion. |
| (Hammel and Galloway 2000) [Europe] | To examine short-term responses to scarcity in the context of medium and long-term historical change | Parish residents in the Northwest Balkans in 18th and 19th Centuries | Mixed methods | Fertility responses in the year of a price shock come to dominate those in the year following, suggesting a shift from contraception to abortion as economic and social conditions apparently worsened and strategies of control intensified. Analysis of monthly responses supports the conjecture based on the annual responses. The shift to the preventive check and strength of the preventive check in the same year as the price shock is unusual in Europe and beyond. |
| (Hu, Bertozzi et al. 2007) [Mexico] | To conduct a cost effectiveness analysis of alternative strategies to reduce maternal mortality and morbidity in Mexico | A computer-based model that simulates the natural history of pregnancy and pregnancy-related complications in a cohort of 15-year-old women followed over their lifetime | An empirically calibrated model that simulates the natural history of pregnancy and pregnancy-related complications | A combined approach that improves access to safe abortion and increases effective coverage for family planning is synergistic in reducing unwanted pregnancies, reducing maternal morbidity and mortality, and increasing health returns for investments using public health dollars. The cost savings from providing these two interventions together, over the long-term, exceeds $11 million per 100,000 women of reproductive age followed over their lifetime. Using current census data from Mexico, the most effective strategy that included enhanced family planning and safe abortion as two of its three main components, would save approximately $116 million on average, over the lifetime of a single birth cohort. The savings in high-marginality states ($49,572,000) would be greater than those with medium marginality ($39,579,000) or lower marginality ($26,530,000). |
| (Hu, Grossman et al. 2010) [Sub-Saharan Africa] | To explore the policy implications of increasing access to safe abortion in Nigeria and Ghana | Women seeking abortion | Decision analytic model | Transitioning from unsafe to safe abortion, regard- less of modality, is the most influential factor on saving both lives and societal costs. For example, provision of safe abortion for 60% of women that would otherwise get an unsafe abortion has a large impact on years of life saved (~11,000 years life saved per 100,000 procedures in Nigeria and ~ 17,500 years life saved per 100,000 procedures in Ghana). Furthermore, if access to medical abortion provides a feasible and acceptable option, such that all women are assured access to safe abortion, years of life gained per 100,000 procedures are more than 18,300 and 29,000 in Nigeria and Ghana, respectively, compared with unsafe abortion. In contrast, a transition in practice pattern from one where 50% of women that would other- wise receive D&C to clinic-based MVA, with- out changing the percentage that pursue un- safe abortion, provides very small incremental health benefits (~3 years life saved per 100,000 procedures) although does save substantial costs. Results underscore the importance of enhancing access to safe abortion in Nigeria, Ghana, and similar countries in sub-Saharan Africa. |
| (Jarlenski, Hutcheon et al. 2017) [United States] | To estimate association between state Medicaid coverage of medically necessary abortion and severe maternal morbidity and in-hospital maternal mortality | Pregnancy-related hospitalizations | Regression analysis | Across both Medicaid and private paid hospitalizations, state Medicaid coverage of medically necessary abortion was associated with an adjusted average of 9.5 per 10,000 fewer cases of severe maternal morbidity, which translates to an average 16% risk reduction. Among women with Medicaid, the unadjusted rate of in-hospital mortality was 9.1 per 100,000 pregnancy-related hospitalizations, which did not differ significantly by state Medicaid abortion coverage status. The average predicted risk of in-hospital maternal mortality was not different among Medicaid-paid hospitalizations in states with or without Medicaid coverage of medically necessary abortion. Likewise, there was no significant difference in in-hospital mortality among private insurance-paid hospitalizations in states with or without Medicaid coverage of medically necessary abortion. |
| (Kelly and Grant 2007) [United States] | Using state-level data, this study analyzes the effects of state policies enacted in the wake of welfare reform on reproductive health | State level abortion rates and birth rates | Regression analysis | The authors find that economic-based incentives have only minor, and inconsistent, influence on statewide rates of abortion and nonmarital births in 2000. Results are consistent with feminist scholarship proposing that noneconomic considerations are more central in women’s decision making about reproduction than economic factors. |
| (Levin, Grossman et al. 2009) [Mexico] | To assess abortion outcomes and costs to the health care system in Mexico City in 2005 at a mix of public and private facilities prior to the legalization of abortion | Hospital staff, administrative records and patients at 3 public hospitals and 1 private clinic in Mexico City | Cost estimates and projections | Increasing access to manual vacuum aspiration and early abortion with misoprostol could reduce government costs by 62%, with potential savings of up to US $1.6 million per year. Reducing complications by improving access to safe services in outpatient settings would further reduce the costs of abortion care, with significant benefits both to Mexico's health care system and women seeking abortion. In Scenario 2, even a modest increase in access to MVA would provide additional cost savings of over US $50,000. Key to these scenarios is that the cost of providing abortion services declines as access increases at smaller public and private health facilities, which can provide abortion care efficiently and at less cost than large hospitals. |
| (Meier and McFarlane 1994) [United States] | Examine whether state family planning expenditures and abortion funding for Medicaid-eligible women affect different kinds of births | State abortion rates | Regression analysis | All other things being equal, an increase of one funded abortion per 1000 women of childbearing age is associated with 0.673 fewer teen births per 1000 teenage women, 0.024 percentage points fewer low-birthweight babies, 0.027 percentage points fewer premature births, and 0.263 percentage points fewer births with late or no prenatal care. Two comments about the size of these coefficients are in order. First, funded abortions are associated with a major drop in births to teen mothers--perhaps as many as 0.67 teen births for every abortion funded. Second, many other impacts of abortion funding or family planning appear modest; however, even small changes in these variables can have a major impact. |
| (Rodriguez, Mendoza et al. 2015) [Colombia] | To compare the costs to the health system of three approaches to the provision of abortion care in Colombia | Three sites in Colombia that were high-volume institutions that offer legal abortions and PAC | Cost analysis | Sensitivity analysis demonstrated that legal abortion would need to cost more than $734 per case for PAC to be a cost-saving strategy. If one assumes a 9% spontaneous abortion rate (based on population level estimates), and if the remainder of PAC cases currently observed were replaced with legal abortion (medical or MVA), the health system would save an additional $163,000 dollars and prevent 16 complications per 1,000 abortions. The health system could save an additional $177,000 (per 1,000 women) from baseline, by replacing D&C with MVA. |
| (Sethe and Murdoch 2013) [United Kingdom] | Consider two clinical procedures, abortion and IVF treatment, which have similar ethical and political sensitivities | Anyone considering abortion or IVF | Literature review | In the UK, the provision of the NHS treatment can be a proxy for social acceptance: Abortion is free on the NHS but women may decide to pay privately to avoid GP referral or waiting times. NHS abortion provision ranges from more than 90% to less than 60% of local demand. From 2002 to 2009, the number NHS funded abortions increased from 78 to 94%. |
| (Sutton 2017) [Argentina] | Explore how several clandestine zones build women's bodies in vital ways to the sovereign power of the State, both in dictatorship and in democracy | Women in Argentina | Meta-analysis | In the case of women who have clandestine induced abortions, women affirm their rights as human beings, but at the cost of their exclusion from the political body. Instead of being able to exercise their rights in the protection of the law, in the field of law and on the margins of institutions. The criminalization of abortion creates a contradiction for the state: on the one hand, the state has an interest in the bodies of individual women in a punitive sense, but on the other hand, it also has a biopolitical interest in a healthy population. |
| (Thomas 2007) [India] | Sets out a conceptual framework to clarify the process by which the demand and supply continuum that underpins the market for reproductive services is sustained by repeated and frequent abortions | Supply and demand for sex-selective abortions | Literature review | By 2005, ultrasound scanning for sex determination had become a Rs.5 billion industry. The upward trend in sex-selective abortions and the flourishing abortion business are linked. The regulatory framework established by the MTP underpins this linkage. The evidence that the sex-selective abortion business is flourishing even after the practice has been criminalized indicates two things: there is a demand for sex-selective abortions and the medical practitioners and abortion seekers are strategically avoiding the law to meet this demand. If the latter does not, then the PCPNDT will criminalize both her and her family. This mutual, strategic, avoidance is not only necessary to sustain the proliferating business of sex-selective abortions but it reveals a fissure between legal ordering or the formal law (which by all accounts has failed) and the underground system of private orderings (in which the sex-selective abortion business flourishes). |
| (Tunc 2008) [United States] | Review the vacuum aspirator's history and why, in less than a decade, electric vacuum suction became American physicians' abortion technology of choice | Americans involved in the provision/receipt of abortion | Historical review | Legalized pregnancy termination inspired many physicians to try new abortion technologies, such as the Karman cannula, which won converts to electrical vacuum aspiration by making it a great deal cheaper and safer. Thus, this critical juncture not only facilitated the diffusion of electrical vacuum suction, but also reinforced the authority of specialized professionals as the sole purveyors of pregnancy termination, and its techniques. |

**References**

Almond, D., L. Edlund and K. Milligan (2013). "Son Preference and the Persistence of Culture: Evidence from South and East Asian Immigrants to Canada." Population and Development Review **39**(1): 75-95.

Ananat, E. O., J. Gruber, P. B. Levine and D. Staiger (2009). "Abortion and Selection." Review of Economics and Statistics **91**(1): 124-136.

Belton, S. and A. Whittaker (2007). "Kathy Pan, sticks and pummelling: Techniques used to induce abortion by Burmese women on the Thai border." Social Science & Medicine **65**(7): 1512-1523.

Benson, J., H. Gebreselassie, M. A. Manibo, K. Raisanen, H. B. Johnston, C. Mhango and B. A. Levandowski (2015). "Costs of postabortion care in public sector health facilities in Malawi: a cross-sectional survey." BMC Health Serv Res **15**: 562.

Benson, J., M. Okoh, K. KrennHrubec, M. A. Lazzarino and H. B. Johnston (2012). "Public hospital costs of treatment of abortion complications in Nigeria." Int J Gynaecol Obstet **118 Suppl 2**: S134-140.

Bullard, K. A., B. L. Shaffer, K. S. Greiner, A. E. Skeith, M. I. Rodriguez and A. B. Caughey (2018). "Twenty-Week Abortion Bans on Pregnancies With a Congenital Diaphragmatic Hernia: A Cost-Effectiveness Analysis." Obstetrics & Gynecology **131**(3): 581-590.

Cheng, Y., Y. Zhou, Y. Zhang, X. Jiang, M. Xi, K. Gan and S. Ren (2012). "Study of knowledge and attitudes on medical abortion among Chinese health providers." International Journal of Gynecology & Obstetrics **118**: S28-S32.

Comendant, R. (2005). "A project to improve the quality of abortion services in Moldova." Reproductive Health Matters **13**(26): 93-100.

Elias, J. J., N. Lacetera, M. Macis and P. Salardi (2017). "Economic Development and the Regulation of Morally Contentious Activities." American Economic Review **107**(5): 76-80.

Erim, D. O., S. C. Resch and S. J. Goldie (2012). "Assessing health and economic outcomes of interventions to reduce pregnancy-related mortality in Nigeria." BMC Public Health **12**: 786.

Fletcher, R. (2000). "National crisis, supranational opportunity: the Irish construction of abortion as a European service." Reproductive Health Matters **8**(16): 35-44.

Foster, D. G., M. A. Biggs, S. Raifman, J. Gipson, K. Kimport and C. H. Rocca (2018a). "Comparison of Health, Development, Maternal Bonding, and Poverty Among Children Born After Denial of Abortion vs After Pregnancies Subsequent to an AbortionHealth, Development, Maternal Bonding, and Poverty Among Children Born After Denial of AbortionHealth, Development, Maternal Bonding, and Poverty Among Children Born After Denial of Abortion." JAMA Pediatrics **172**(11): 1053-1060.

Foster, D. G., M. A. Biggs, L. Ralph, C. Gerdts, S. Roberts and M. M. Glymour (2018b). "Socioeconomic Outcomes of Women Who Receive and Women Who Are Denied Wanted Abortions in the United States." American Journal of Public Health **108**(3): 407-413.

Haas-Wilson, D. (1996). "The Impact of State Abortion Restrictions on Minors' Demand for Abortions." Journal of Human Resources **31**(1): 140-158.

Hammel, E. A. and P. R. Galloway (2000). "Structural and Behavioural Changes in the Short Term Preventive Check in the Northwest Balkans in the 18th and 19th Centuries." European Journal of Population / Revue Européenne de Démographie **16**(1): 67-108.

Hu, D., S. M. Bertozzi, E. Gakidou, S. Sweet and S. J. Goldie (2007). "The costs, benefits, and cost-effectiveness of interventions to reduce maternal morbidity and mortality in Mexico." PLoS One **2**(8): e750.

Hu, D., D. Grossman, C. Levin, K. Blanchard, R. Adanu and S. J. Goldie (2010). "Cost-effectiveness analysis of unsafe abortion and alternative first-trimester pregnancy termination strategies in nigeria and ghana." African Journal of Reproductive Health **14**(2): 85-103.

Jarlenski, M., J. A. Hutcheon, L. M. Bodnar and H. N. Simhan (2017). "State Medicaid Coverage of Medically Necessary Abortions and Severe Maternal Morbidity and Maternal Mortality." Obstetrics and Gynecology **129**(5): 786-794.

Kelly, K. and L. Grant (2007). "State Abortion and Nonmarital Birthrates in the Post-Welfare Reform Era: The Impact of Economic Incentives on Reproductive Behaviors of Teenage and Adult Women." Gender and Society **21**(6): 878-904.

Levin, C., D. Grossman, K. Berdichevsky, C. Diaz, B. Aracena, S. G. Garcia and L. Goodyear (2009). "Exploring the costs and economic consequences of unsafe abortion in Mexico City before legalisation." Reproductive Health Matters **17**(33): 120-132.

Meier, K. J. and D. R. McFarlane (1994). "State family planning and abortion expenditures: Their effect on public health." American Journal of Public Health **84**(9): 1468-1472.

Rodriguez, M. I., W. S. Mendoza, C. Guerra-Palacio, N. A. Guzman and J. E. Tolosa (2015). "Medical abortion and manual vacuum aspiration for legal abortion protect women’s health and reduce costs to the health system: findings from Colombia." Reproductive Health Matters **22**: 125-133.

Sethe, S. and A. Murdoch (2013). "Comparing the Burden: What Can We Learn by Comparing Regulatory Frameworks in Abortion and Fertility Services?" Health Care Analysis **21**(4): 338-354.

Sutton, B. (2017). "Zonas de clandestinidad y “nuda vida:” Mujeres, cuerpo y aborto." Estudos Feministas **25**(2): 889-902.

Thomas, D. (2007). "Abortion Law and the Unregulated Business of Female Sex-Selective Abortions in India." Web Journal of Current Legal Issues(5).

Tunc, T. E. (2008). "Designs of devices: the vacuum aspirator and American abortion technology." Dynamis **28**: 353-376.
